# Niclosamide: CRL4^AMBRA1^ mediated degradation of cyclin D1 following mitochondrial membrane depolarization†

**DOI:** 10.1039/d5md00054h

**Published:** 2025-05-06

**Authors:** Seemon Coomar, Jessica A. Gasser, Mikołaj Słabicki, Katherine A. Donovan, Eric S. Fischer, Benjamin L. Ebert, Dennis Gillingham, Nicolas H. Thomä

**Affiliations:** a Friedrich Miescher Institute for Biomedical Research Fabrikstrasse 24 Basel 4056 Switzerland; b Broad Institute of MIT and Harvard Cambridge MA 02142 USA; c Department of Medical Oncology, Dana-Farber Cancer Institute Boston MA 02115 USA; d Krantz Family Center for Cancer Research, Massachusetts General Hospital Cancer Center Charlestown MA 02129 USA; e Department of Cancer Biology, Dana-Farber Cancer Institute 450 Brookline Ave. Boston MA 02215 USA; f Department of Biological Chemistry and Molecular Pharmacology, Harvard Medical School 240 Longwood Ave. Boston MA 02215 USA; g Dana-Farber Cancer Institute Boston MA 02115 USA; h Department of Chemistry, University of Basel Basel 4056 Switzerland; i Swiss Institute for Experimental Cancer Research (ISREC) EPFL, Station 19 Lausanne 1015 Switzerland nicolas.thoma@epfl.ch

## Abstract

Targeted protein degradation has emerged as a promising approach in drug discovery, utilizing small molecules like molecular glue degraders to harness the ubiquitin-proteasome pathway for selective degradation of disease-driving proteins. Based on results from proteomics screens we investigated the potential of niclosamide, an FDA-approved anthelmintic drug with a 50 year history in treating tapeworm infections, as a molecular glue degrader targeting the proto-oncogene cyclin D1. Proteomics screens in HCT116 colon carcinoma and KELLY neuroblastoma cells, found that niclosamide induces rapid cyclin D1 degradation through a mechanism involving the ubiquitin-proteasome pathway. A genetic CRISPR screen identified the E3 ligase CRL4^AMBRA1^ as a key player in this process. Structure–activity relationship studies highlighted critical features of niclosamide necessary for cyclin D1 degradation, demonstrating a correlation between mitochondrial membrane potential (MMP) disruption and cyclin D1 downregulation. Notably, various mitochondrial uncouplers and other compounds with similar drug sensitivity profiles share this correlation suggesting that MMP disruption can trigger cyclin D1 degradation, and that the cellular signal driving the degradation differs from previously described mechanism involving CRL4^AMBRA1^. Our findings underscore the complexities of proteostatic mechanisms and the multitude of mechanisms that contribute to degrader drug action.

## Abstract 2

Niclosamide, an FDA-approved anthelmintic drug, known to uncouple the mitochondria induces cyclin D1 degradation *via* CRL4^AMBRA1^. We find a striking correlation between the two processes and that it is observed amongst various compounds.

## Graphical abstract

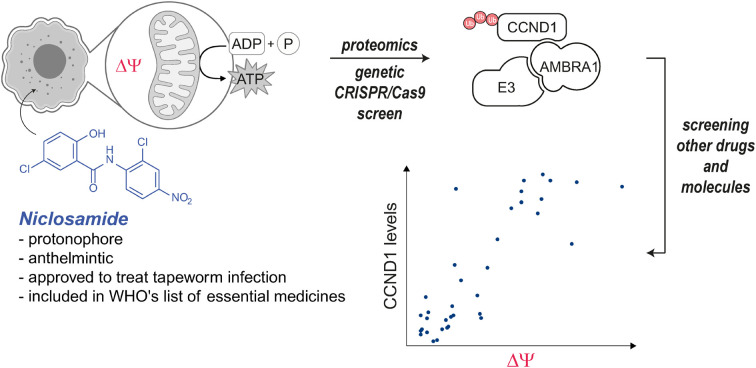

## Introduction

Small molecules that induce proximity to yield therapeutically beneficial ternary complexes of disease-relevant proteins are an emerging topic in drug discovery.^1,2^ The most frequent use of the concept has been in the field of targeted protein degradation (TPD), which has generated considerable clinical and commercial interest.^3,4^ The field of TPD gained considerable momentum after the mechanistic understanding of immunomodulatory drugs (IMiDs). They degrade the transcription factors (TFs) Ikaros and Aiolos and drive anti-myeloma activity in ubiquitin-dependent manner, which catalysed research into the development of molecules that can degrade disease-driving proteins.^5^ In the case of IMiDs such as lenalidomide (Revlimid), small molecules induce the complex formation between the E3 ligase adapter protein CRBN and the TFs leading to the ubiquitination and consequential degradation of the latter.^6,7^ Structural studies suggested that the molecules could be used as anchors to design bifunctional molecules, known as proteolysis targeting chimeras (PROTACs) that could engage CRBN and a protein of interest that would be degraded by the ubiquitin proteasome system (UPS).^8^ Whereas a rational design is possible for PROTACs, the discovery of small molecules which induce a novel interaction between an E3 ligase and a protein, so-called molecular glue degraders (MGDs) is more challenging as the binding partners are unknown *a priori*.^9–11^

A typical workflow in the discovery process of a MGD begins with treating cells for a short duration with a compound, followed by global proteomics experiments. If a target of interest is selectively degraded, follow-up studies are conducted to confirm that the degradation occurs *via* the UPS by inhibiting key components of the pathway. Subsequently, various approaches can be employed to identify components involved in ternary complex formation, such as the E3 ligase or the target protein interface. One effective method is to perform CRISPR/Cas9-based genetic screens using a focused guide library containing UPS components.^12^

Anthelmintic drugs were historically discovered by repurposing known chemical entities^13^ using screens in whole parasites as preclinical disease models. One notable example is niclosamide, first discovered in 1953 at Bayer through a screening and hit optimization project targeting *Biomphalaria glabrata*, a snail intermediate host of the parasite *Schistosoma mansoni.*^14^ Niclosamide was marketed as Bayluscide in 1959. Later found effective against human tapeworms, it gained FDA approval in 1982 and is now a WHO essential medicine.^15^ Since then, like other anthelmintic drugs, it has been evaluated for various indications, including different types of cancer^15^ and infectious diseases such as SARS-CoV-2, undergoing multiple clinical trials. Despite its poor systemic bioavailability, its approved use against intestinal parasites has led to trials for colorectal cancer, prostate cancer, and recently a phase I trial against acute myeloid leukemia (http://clinicaltrials.gov, NCT05188170).^16,17^ While multiple molecular targets have been proposed, whether there is a primary mode of action explaining the pleiotropic effects of the drug remains unclear.^18^

On the basis of global quantitative proteomics screening results, we investigated niclosamide as a potential molecular glue degrader (MGD) of the proto-oncogene cyclin D1 (CCND1). We first conducted a CRISPR screen to identify members of the UPS that are involved. Subsequent structure–activity relationship assays on the molecule revealed a striking correlation between their effect on cyclin D1 levels and the impact on the mitochondria membrane potential (MMP). This correlation extended to other known mitochondria uncouplers and even seemingly unrelated molecules with drug sensitivity profiles similar to niclosamide. Our research highlights the multitude of complex cellular signalling and stress pathways that can lead to protein degradation.

## Results and discussion

### Niclosamide leads to cyclin D1 degradation

**Fig. 1:**
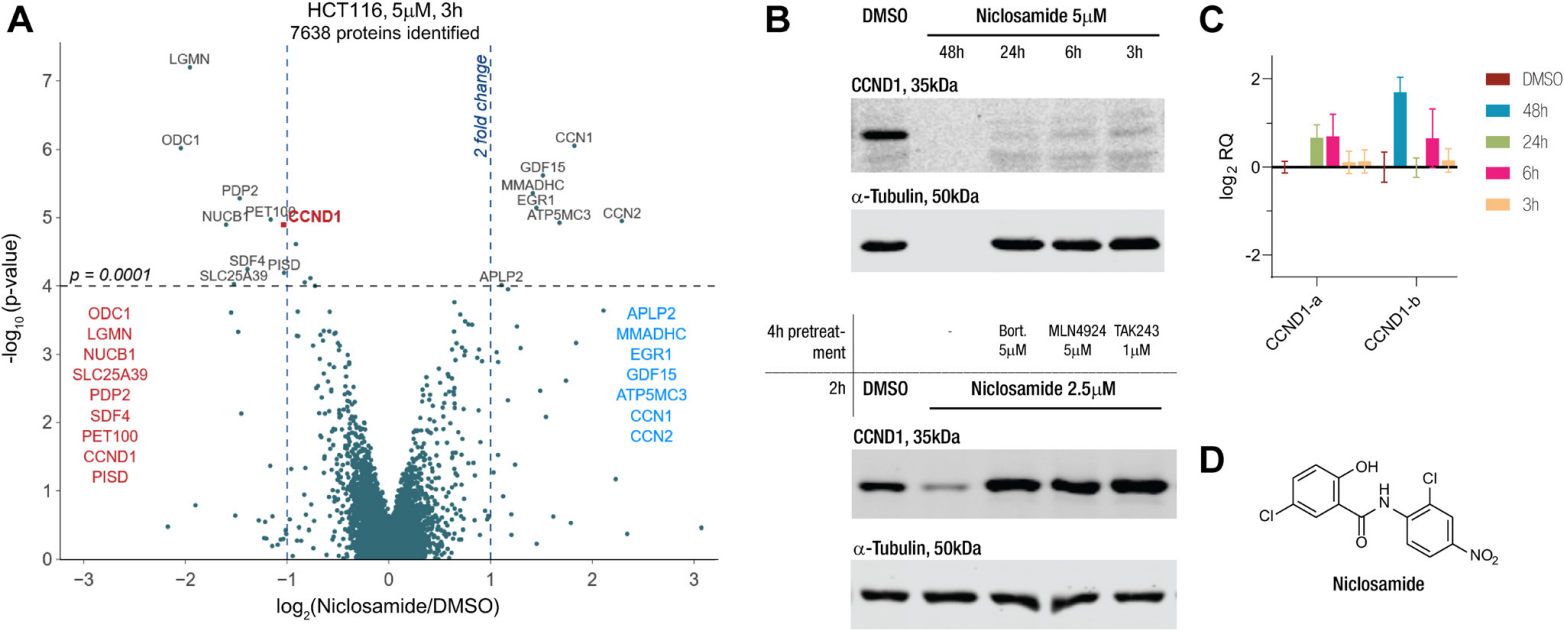
Niclosamide induces proteostatic degradation of CCND1 in HCT116 cells. A) Change in protein levels relative to vehicle treatment (DMSO) in HCT116 cells treated with niclosamide at 5 μM for 3 h quantified by TMT labelling and LC MS/MS analysis *versus p*-value. B) (top) Western blot of anti-CCND1 and anti-tubulin of HCT116 cells treated with vehicle (DMSO), niclosamide at different 5 μM for different time points and (bottom) pre- and co-treated with bortezomib (5 μM), MLN4924 (5 μM) and TAK243 (1 μM). High cytotoxicity observed at 48 h treatment time. C) Relative quantification (RQ) amplicon levels from primer pair a normalized to vehicle treatment (DMSO) in HCT116 cells treated with the niclosamide at 5 μM for the indicated times. Values represent mean −ΔΔCt values ± SEM from triplicates (*n* = 3). D) Structure of niclosamide.

Our proteomics screens in HCT116 colon carcinoma and KELLY neuroblastoma cells identified niclosamide as a drug that rapidly induced changes to the proteome (Fig. 1A and S1A†). Particularly, we became interested in its potential to degrade cyclin D1 *via* an apparent MGD mechanism. We treated HCT116 with niclosamide and checked the protein and transcript levels of cyclin D1 *via* immunoblotting and qPCR (Fig. 1B and C). In line with the proteomics results, we saw rapid protein degradation while the transcript levels remained unchanged. Co-treatment with E1 inhibitor TAK243, NEDD8 inhibitor MLN4924, and proteasome inhibitor bortezomib rescued protein levels, indicating the involvement of a cullin E3 ligase (Fig. 1B). These findings link niclosamide to cyclin D1 degradation through a UPS-dependent mechanism, potentially acting as an MGD.

### Cyclin D1 degradation is driven by CRL4^AMBRA1^

**Fig. 2:**
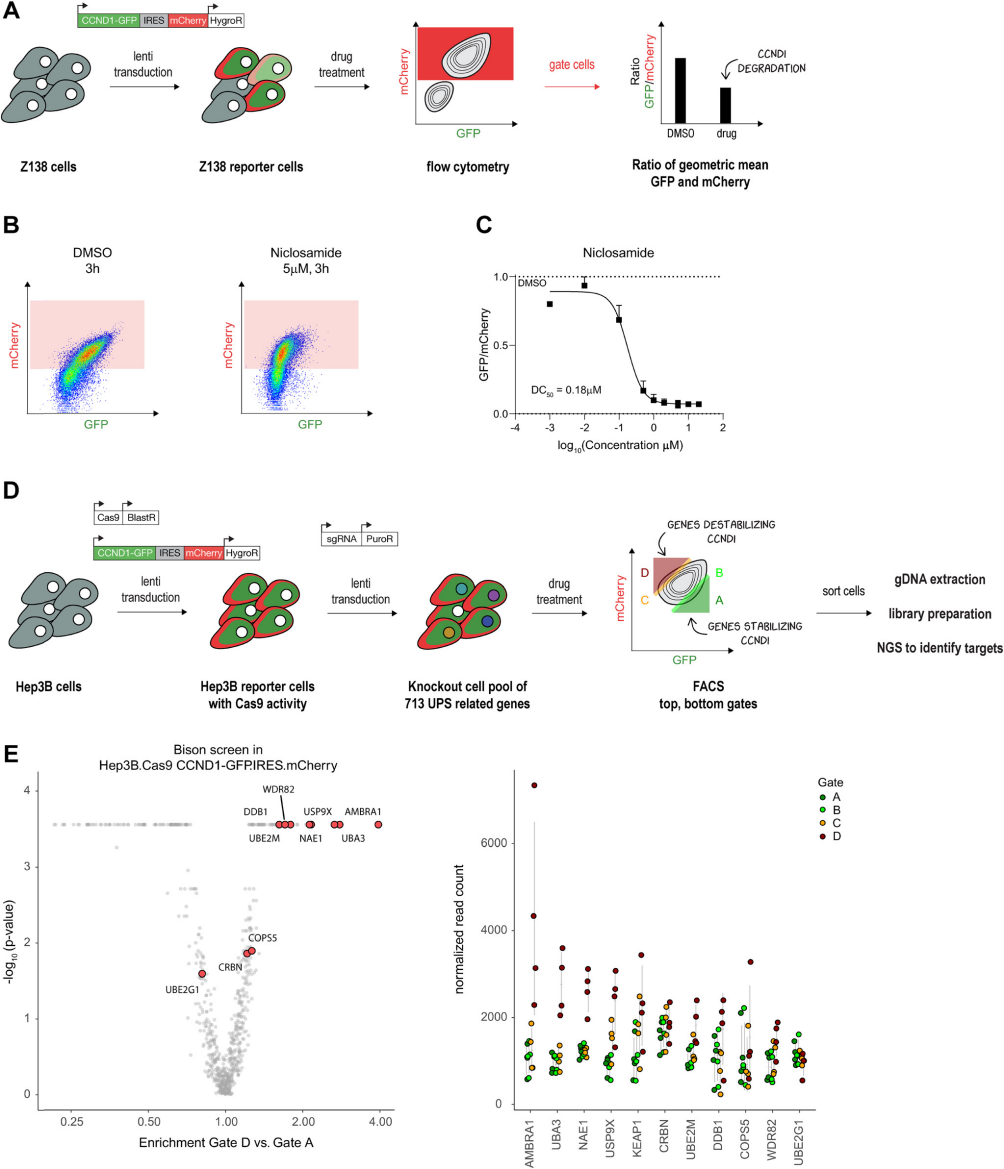
CCND1 reporter cell lines capture degradation activity. A) Set-up towards establishing the fluorescence-based reporter system for quantifying CCND1 protein levels in Z138 cells. B) The population of Z138 reporter cells (Z138 CCND1-GFP.IRES.mCherry) gated by flow cytometry and used for quantification of CCND1. C) Dose response curve of CCND1 levels relative to vehicle (DMSO) treatment in the reporter cells treated with niclosamide for 3 h. Values represent the ratio of the geometric mean of GFP and mCherry values in the gated population. D) Workflow of setting up a CCND1 reporter in Hep3B cell with Cas9 activity and performing a FACS based genetic screen of members of the UPS which can rescue drug induced CCND1 degradation. E) Genes enriched in a FACS based genetic CRISPR screen (Bison library) rescuing niclosamide induced CCND1 degradation in Hep3B.Cas9.CCND1-GFP.IRES.mCherry reporter cells. The gates used are shown in D) and each contain 5% of the population shown. Gate D contains cells with gene KOs that stabilize CCND1, whereas gate A contains cells with gene KOs that destabilize CCND1.

Intrigued by these results, we sought to identify the E3 ligase necessary for niclosamide-induced degradation of cyclin D1. We employed a FACS-based genetic CRISPR screen, previously successful in our studies.^19,20^ We established a stable cyclin D1 two-color reporter system in Z-138 acute lymphoblastic leukemia cells, using a construct with a cyclin D1-GFP fusion and an independently translated mCherry for comparative quantification (Fig. 2A). After confirming that the reporter system response mirrored that of endogenous protein (Fig 2B and C), we proceeded with genetic screens.

Due to low Cas9 activity in Z-138 cells, we switched to Cas9-bearing Hep3B hepatoma cells. We transduced the Hep3B reporter cells with the Bison library (a sgRNA pool targeting 713 UPS-related genes) and analyzed cells showing no cyclin D1 degradation upon compound treatment (Fig. 2D). We saw an enrichment for cells lacking AMBRA1, the substrate adaptor protein in the E3 ligase CRL4^AMBRA1^, which controls endogenous cyclin D1 levels^21–23^ or one of the members of the dimeric UBA3-NAE1 NEDD8 E1 enzyme (Fig. 2E). The importance of AMBRA1 in niclosamide-mediated degradation of cyclin D1 was validated in HCT-116^AMBRA−/−^ cells where no cyclin D1 degradation was observed within the short treatment time (Fig. S1B†). This suggested that niclosamide either acts as a molecular glue enhancing the AMBRA1–cyclin D1 interaction or that cyclin D1 degradation is part of a cellular response to niclosamide treatment.

### SAR studies reveal distinct features of niclosamide required for cyclin D1 degradation

To identify any induced neomorphic interface involving niclosamide and leading to cyclin D1 degradation, we conducted a structure activity relationship (SAR) study. We aimed to find a site on niclosamide that wouldn't affect the interaction interface, enabling probe design (*e.g.*, for photoaffinity labeling) to elucidate any formed complex. We investigated electronic and steric demands of the two phenyl ring cores by introducing various functional groups and using cyclin D1 degradation as a phenotypic readout.

**Fig. 3:**
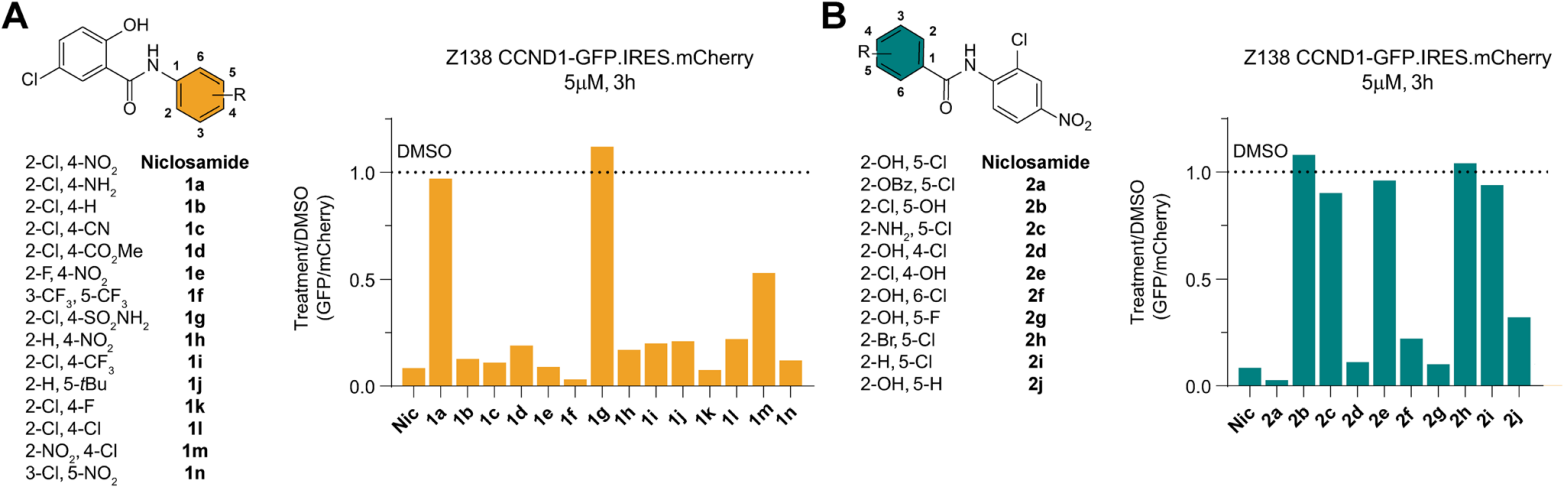
Structure activity study of niclosamide towards CCND1 degradation. A) Molecules probing the nitroarene ring of niclosamide. Change in CCND1 levels relative to vehicle (DMSO) treatment in the reporter cells treated at a concentration of 5 μM for 3 h. Values represent the ratio of the geometric mean of GFP and mCherry values in the gated population. B) As for A) but for molecules probing the salicylamide ring.

We found that the nitroarene ring was relatively tolerant to changes, provided the ring remained electron-withdrawing to some extent. Even the inductive *tert*-butyl group was tolerated, but increasing electron-donating properties by changing to an aniline or a pyridine abolished all degradation activity (Fig. 3A and S2A†). The loss of degradation of the arylsulfonamide was attributed to possible steric clashes or off-target effects, which were not further investigated. Altering the type and position of the halide on the salicylamide ring revealed that only the acidic^24,25^ hydroxy group *ortho* to the amide bond was necessary to induce cyclin D1 degradation (Fig. 3B and S2A†).

We also examined the importance of the connectivity between the two arene rings. Inverting the amide bonds in one molecule and increasing flexibility while reducing electronic conjugation by introducing an extra CH_2_ in another molecule (Fig. S2B and D†) resulted in no induced degradation, highlighting the crucial role of the amide bond. To mask the charge of a deprotonated hydroxy group on the salicylamide ring, we bridged it *via* a carbamate to the amide bond. Although this molecule was active, we could not rule out the possibility of it being a prodrug hydrolyzed to niclosamide. Taken together these results emphasized the importance of the acidic proton on the salicylamide ring, which has been previously associated with mitochondrial depolarization.^26^ This led us to investigate a potential link between this proton shuttling to the mitochondria and the observed effects on cyclin D1 degradation.

### Mitochondrial depolarization correlates with cyclin D1 degradation

**Fig. 4:**
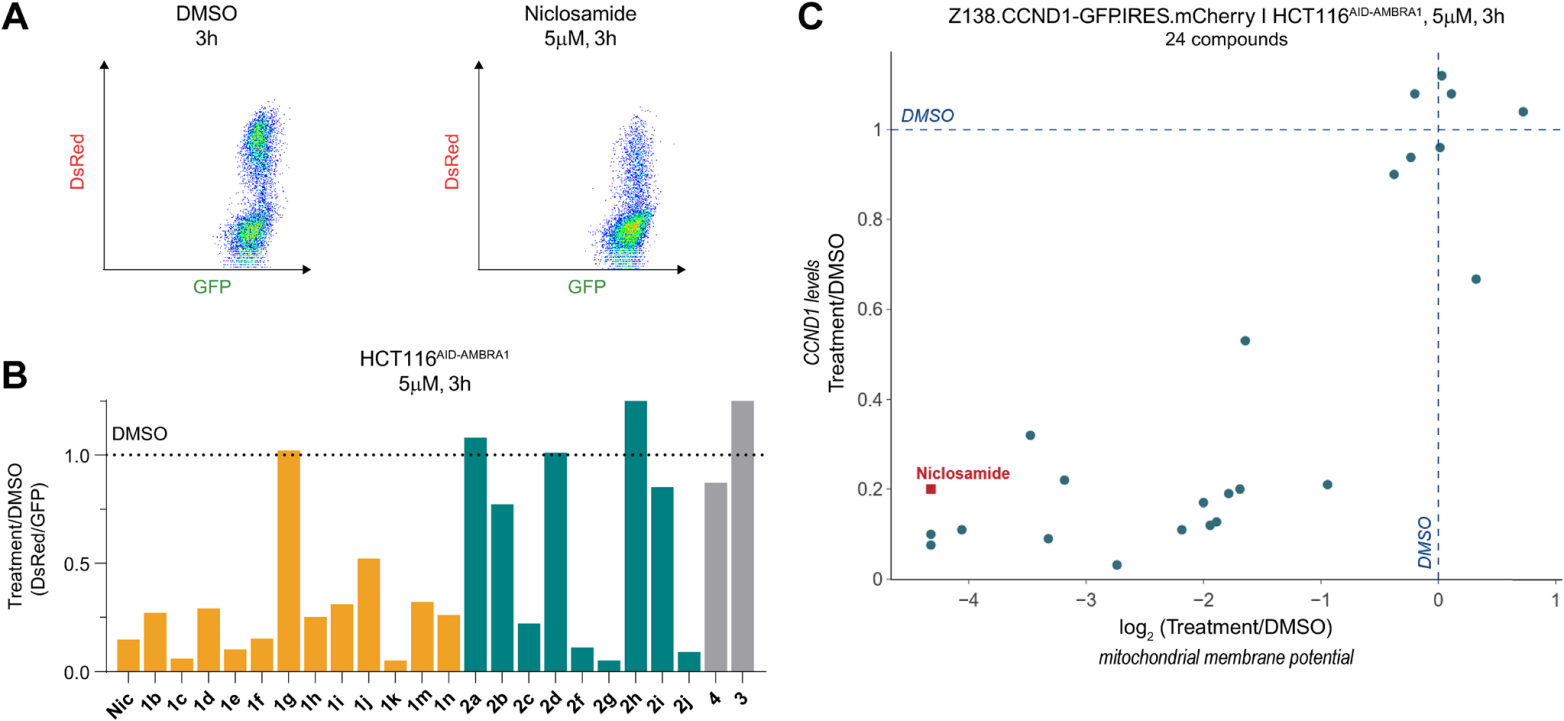
Niclosamide induced CCND1 degradation correlates with mitochondria depolarization. A) Mitochondrial membrane potential (MMP) quantified using flow cytometry in HCT116^AID-AMBRA1^ cells stained with JC-1. B) Change in MMP relative to vehicle (DMSO) treatment in HCT116^AID-AMBRA1^ cells treated at a concentration of 5 μM for 3 h. Values represent the ratio of the geometric mean of GFP and DsRed values of all the measures cells. C) Change in CCND1 levels relative to vehicle (DMSO) quantified in reporter cells (Z138.CCND1-GFP.IRES.mCherry) treated with the compounds listed in B) and plotted against the change in MMP measured in HCT116^AID-AMBRA1^ cells treated with the same compounds at a concentration of 5 μM for 3 h.

To obtain a quantitative comparison of degradation and MMP, we employed JC-1, a cationic dye that exhibits green fluorescence as a monomer but red fluorescence upon mitochondrial accumulation, to assess MMP (Fig. 4A). Hence the ratio of the observed red/green fluorescence is used to evaluate mitochondrial health, where for instance a depolarization reduces the red signal relative to the green signal.

We measured MMP in HCT116^AID-AMBRA1^ cells (Fig. 4B), where an auxin-inducible degron was knocked into the endogenous AMBRA1. This allowed us to induce rapid AMBRA1 degradation and observe its impact on the observed phenotype (Fig. S3A and B†). Together with our measurements in cyclin D1 reporter cells we could use the ratiometric green/red fluorescence readouts to simultaneously evaluate the relationship between cyclin D1 degradation, MMP changes, and the role of AMBRA1 in these processes.

Among the molecules used in the SAR study, we observed a striking correlation between the extent of cyclin D1 degradation and mitochondrial depolarization (Fig. 4C) when cells were treated with a concentration of 5 μM for 3 hours. Notably, induced degradation of AMBRA1 had no effect on depolarization but rescued niclosamide-induced cyclin D1 degradation, as expected.

### Mitochondria depolarizers and other drugs induce CCND1 degradation

**Fig. 5:**
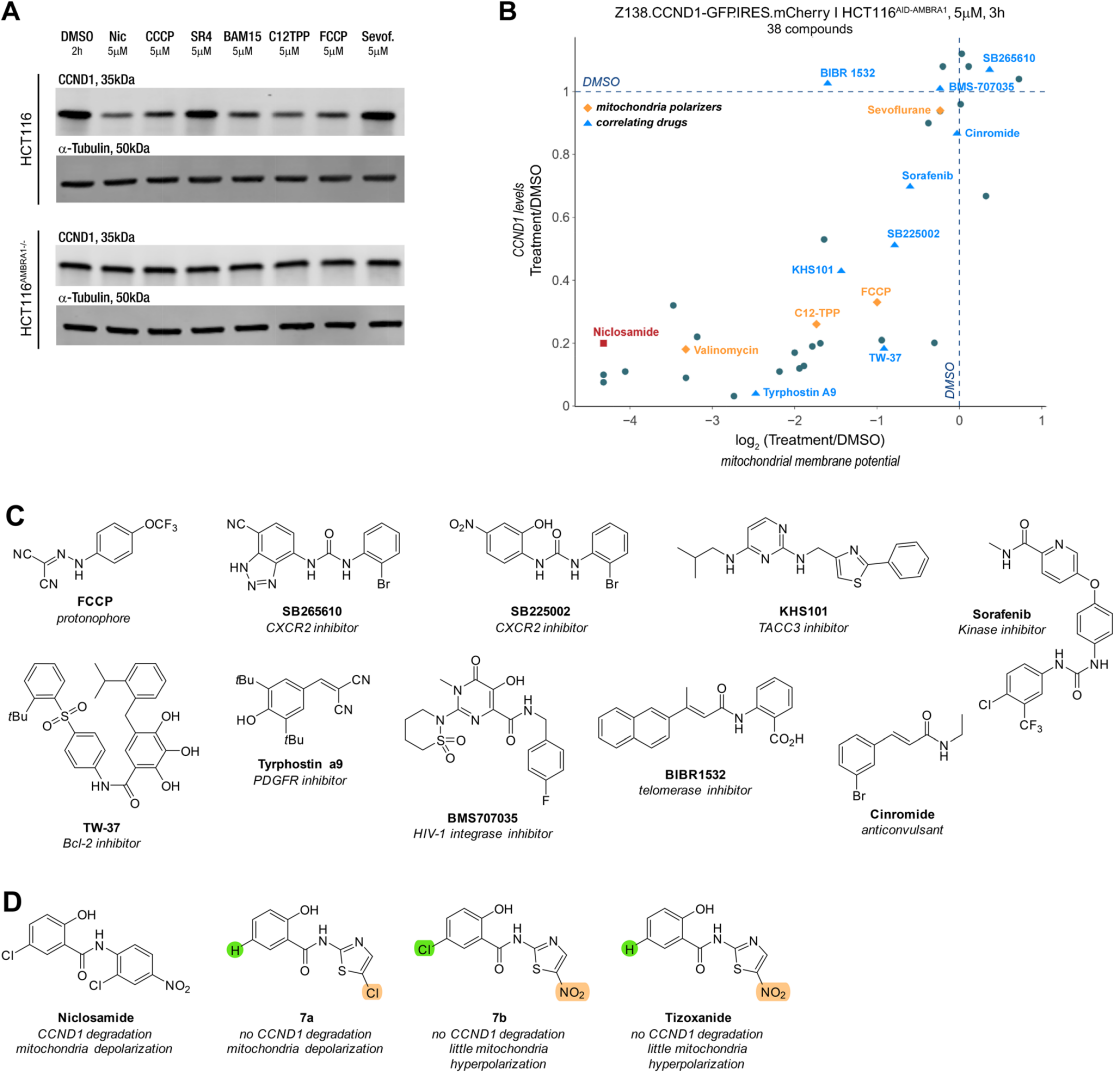
Mitochondria depolarizers and other molecules show same correlation between CCND1 and mitochondrial membrane potential. A) Western blot of anti-CCND1 and anti-tubulin of HCT116 and AMBRA1 KO HCT116^AMBRA1−/−^ cells treated with vehicle (DMSO), niclosamide, CCCP, SR4, BAM15, C12TPP, FCCP and sevoflurane at 5 μM for 2 h. B) Change in CCND1 levels relative to vehicle (DMSO) quantified in reporter cells (Z138.CCND1-GFP.IRES.mCherry) treated with the compounds listed in B) and plotted against the change in mitochondrial membrane potential measured in HCT116^AID-AMBRA1^ cells treated with the same compounds at a concentration of 5 μM for 3 h. C) Structure of the protonophore CCCP and molecules that correlated with niclosamide drug sensitivity according to the AUC secondary screen in PRISM dataset. D) Structures of niclosamide, tizoxanide and other closely structurally related molecules.

To test whether the observed correlation extended to other reported mitochondrial depolarizers with different modes of action, we examined the protonophores CCCP, FCCP, BAM15, the mitochondrial membrane-targeting C12 TPP, SR4,^27^ the anaesthetic sevoflurane^28,29^ and the ionophore valinomycin. To our surprise, all these molecules induced potent cyclin D1 degradation and aligned with the trend seen with the previous series of molecules (Fig. 5A and B). Inspired by our findings, we explored whether other molecules correlating with niclosamide in publicly available drug sensitivity datasets (PRISM, CTRP, accessible on http://depmap.org) exhibited a similar phenotype. Among the top correlates were inhibitors of the anti-apoptotic proteins Bcl-2 and Mcl-1 located on mitochondria, as well as a telomerase inhibitor (BIBR15322) and molecules like KHS101, which targets the cell growth-promoting TACC3, and tyrphostin A9, a tyrosine kinase receptor PDGFR inhibitor. Notably, the correlation between cyclin D1 degradation and mitochondrial depolarization was maintained among the active molecules (Fig. 5B).

While some molecules arguably contained acidic protons, this was not a common structural feature across all compounds (Fig. 5C). Notably, compounds like tyrphostin A9 and KHS-101, whose reported targets do not suggest disruption of mitochondrial function, have been shown to induce cytotoxicity through this mechanism.^30,31^ Interestingly, aside from the lack of niclosamide induced cyclin D1 degradation in HCT116^AMBRA1−/−^ cells, there was no increase in phosphorylation of threonine 286,^23,32–34^ a key PTM for the nuclear export and ubiquitin-mediated degradation (Fig. S3D†).

Even though we aimed to find a molecule that induced cyclin D1 degradation *via* a MGD type mechanism, the chemically diversity of the compounds (Fig. S3E†), which all serve as mitochondrial depolarizers, render it unlikely that these serve as direct MGDs to CRL4^AMBRA1^. Instead, this suggests an indirect mechanism, whereby disruption of the MMP results in the downregulation of cyclin D1 with the help of AMBRA1 and the UPS *via* an interaction that appears distinct from the threoine 286 site used for canonical proteostasis.^21^

### Disrupting mitochondria membrane potential is necessary but not sufficient for cyclin D1 downregulation

It is worth noting that the ionophore nanchangmycin, an antibiotic known to disrupt endocytic uptake of viruses and increase cytosolic Ca^2+^ concentration,^35–37^ showed hyperpolarization and low cyclin D1 levels (Fig. S3C†). The prodrug nitazoxanide of the anti-helminthic tizoxanide, on the other hand, has been reported to depolarize mitochondria.^38,39^ Due to structural similarities with niclosamide, we tested molecular variants of tizoxanide. Under our assay conditions, Tizoxanide and its analogue 7a induced slight mitochondrial hyperpolarization, while 7b led to pronounced depolarization. The molecular variants suggest that the nitro group on the thiazole may influence how the molecule affects MMP but more strikingly, despite structural similarities to niclosamide and sign of depolarisation, neither of the molecules altered cyclin D1 levels (Fig. S3C†). These observations indicate that disrupting MMP is necessary but not sufficient to induce cyclin D1 degradation.

## Conclusion

The rapid downregulation of cyclin D1 upon niclosamide treatment and its recovery with UPS inhibition presented a potential case for a MGD mode of action, with our targeted genetic screens identifying the endogenous ligase CRL4^AMBRA1^ responsible for cyclin D1 degradation. Given the short half-life of cyclin D1 (ref. 32) we briefly considered a mechanism involving post-transcriptional down-regulation through reduced translation; yet finding that niclosamide-mediated degradation is rescued by knock-out or degradation of AMBRA1 renders this unlikely. Our findings firmly placed AMBRA1 in a pathway mediating cyclin D1 degradation following niclosamide induced membrane depolarization. Additionally, we found that molecules with cytotoxic profiles similar to niclosamide also exhibited both degradation and depolarization phenotypes. Yet the absence of cyclin D1 degradation by tizoxanide and related molecules suggested that the disruption of MMP alone was also not sufficient to induce degradation. Given the diversity of chemical structures inducing downregulation, it is unlikely that niclosamide acts directly as the MGD involved in such an interaction. Given the observed polypharmacology of niclosamide we believe that the poor bioavailability of the orally administered drug is key in the lack of toxicity in humans as opposed to tapeworms where the suggested mechanism is indeed the uncoupling of oxidative phosphorylation.^26,33,34^

**Fig. 6:**
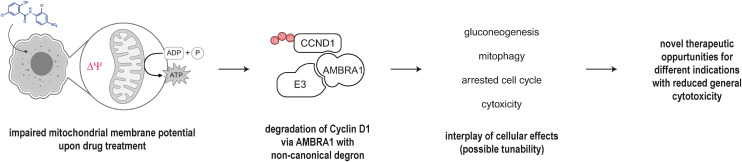
Polypharmacology of niclosamide.

Cyclin D1, in addition to its cell cycle regulatory role, has been directly implicated in glucose metabolism.^40–42^ AMBRA1, in turn, regulates cyclin D1 levels and plays a crucial role in mitophagy induced *via* MMP disruption by translocating to the mitochondria and amongst others interacting with LC3 or parkin.^43–45^ A cell with a compromised MMP, as triggered by a protonophore, is forced to increasingly rely on gluconeogenesis to meet ATP demands anaerobically. Loss of hepatic cyclin D1 similarly leads to increased gluconeogenesis illustrating the direct link between cyclin D1 and mitochondrial integrity. Instead of a direct MGD mechanism, we hypothesize that niclosamide, and other molecules described herein, exploit the physiological circuitry linking MMP and AMBRA1 to induce the degradation of cyclin D1 (Fig. 6). Other examples for small molecule degraders indirectly impacting protein levels by supercharging endogenous pathways have recently been described.^46^ While the majority of mitochondrial depolarizers are toxic and unsuitable as therapeutics, we show that clinically approved drugs can impact the mitochondrial potential and believe that within this window of MMP disruption, derivatives can be made that exhibit superior degradation abilities for cyclin D1 with potentially less unselective toxicity. This is turn would potentially open new potential therapeutic avenues for cyclin D1 degradation while mitigating broad toxicity should niclosamide prove a viable option in future clinical trials.

## Data availability

The proteomics data supporting this article have been included as part of the ESI.† This study was carried out using publicly available data from http://depmap.org. All codes and data analysis pipelines have been published and are stated in the corresponding experimental section.

## Conflicts of interest

N. H. T. is a founder and shareholder of Zenith Therapeutics as well a consultant to Ridgeline Discovery and Red Ridge Bio. B. L. E. has received research funding from Novartis and Calico. He has received consulting fees from Abbvie. He is a member of the scientific advisory board and shareholder for Neomorph Inc., TenSixteen Bio, Skyhawk Therapeutics, and Exo Therapeutics. E. S. F. is a founder, scientific advisory board (SAB) member, and equity holder of Civetta Therapeutics, Proximity Therapeutics, Stelexis Biosciences, and Neomorph, Inc. (also board of directors). He is an equity holder and SAB member for Avilar Therapeutics, Photys Therapeutics, and Ajax Therapeutics and an equity holder in Lighthorse Therapeutics, CPD4, and Anvia Therapeutics. E. S. F. is a consultant to Novartis, EcoR1 capital, Odyssey and Deerfield. The Fischer lab receives or has received research funding from Deerfield, Novartis, Ajax, Interline, Bayer and Astellas. K. A. D. receives or has received consulting fees from Neomorph Inc and Kronos Bio. M. S. has received research funding from Calico Life Sciences LLC.

## Supplementary Material

MD-OLF-D5MD00054H-s001

MD-OLF-D5MD00054H-s002

MD-OLF-D5MD00054H-s003

MD-OLF-D5MD00054H-s004

MD-OLF-D5MD00054H-s005

MD-OLF-D5MD00054H-s006

## References

[cit1] Gerry C. J., Schreiber S. L. (2020). Unifying principles of bifunctional, proximity-inducing small molecules. Nat. Chem. Biol..

[cit2] Schreiber S. L. (2024). Molecular glues and bifunctional compounds: Therapeutic modalities based on induced proximity. Cell Chem. Biol..

[cit3] Bekes M., Langley D. R., Crews C. M. (2022). PROTAC targeted protein degraders: the past is prologue. Nat. Rev. Drug Discovery.

[cit4] Arnold C. (2024). PROTAC protein degraders to drug the undruggable enter phase 3 trials. Nat. Med..

[cit5] Oleinikovas V. (2024). *et al.*, From Thalidomide to Rational Molecular Glue Design for Targeted Protein Degradation. Annu. Rev. Pharmacol. Toxicol..

[cit6] Kronke J. (2014). *et al.*, Lenalidomide causes selective degradation of IKZF1 and IKZF3 in multiple myeloma cells. Science.

[cit7] Ito T. (2010). *et al.*, Identification of a primary target of thalidomide teratogenicity. Science.

[cit8] Fischer E. S. (2014). *et al.*, Structure of the DDB1-CRBN E3 ubiquitin ligase in complex with thalidomide. Nature.

[cit9] Garber K. (2024). The glue degraders. Nat. Biotechnol..

[cit10] Dewey J. A. (2023). *et al.*, Molecular Glue Discovery: Current and Future Approaches. J. Med. Chem..

[cit11] Robinson S. A., Co J. A., Banik S. M. (2024). Molecular glues and induced proximity: An evolution of tools and discovery. Cell Chem. Biol..

[cit12] Slabicki M. (2020). *et al.*, The CDK inhibitor CR8 acts as a molecular glue degrader that depletes cyclin K. Nature.

[cit13] Pink R. (2005). *et al.*, Opportunities and challenges in antiparasitic drug discovery. Nat. Rev. Drug Discovery.

[cit14] Andrews P., Thyssen J., Lorke D. (1982). The biology and toxicology of molluscicides, Bayluscide. Pharmacol. Ther..

[cit15] Chen W. (2018). *et al.*, Niclosamide: Beyond an antihelminthic drug. Cell. Signalling.

[cit16] Laudisi F. (2020). *et al.*, Repositioning of Anthelmintic Drugs for the Treatment of Cancers of the Digestive System. Int. J. Mol. Sci..

[cit17] Seo J. I., Jin G. W., Yoo H. H. (2024). Pharmacokinetic considerations for enhancing drug repurposing opportunities of anthelmintics: Niclosamide as a case study. Biomed. Pharmacother..

[cit18] Li Y. (2014). *et al.*, Multi-targeted therapy of cancer by niclosamide: A new application for an old drug. Cancer Lett..

[cit19] Slabicki M. (2020). *et al.*, Small-molecule-induced polymerization triggers degradation of BCL6. Nature.

[cit20] Zou C. (2023). *et al.*, The human E3 ligase RNF185 is a regulator of the SARS-CoV-2 envelope protein. iScience.

[cit21] Simoneschi D. (2021). *et al.*, CRL4(AMBRA1) is a master regulator of D-type cyclins. Nature.

[cit22] Chaikovsky A. C. (2021). *et al.*, The AMBRA1 E3 ligase adaptor regulates the stability of cyclin D. Nature.

[cit23] Maiani E. (2021). *et al.*, AMBRA1 regulates cyclin D to guard S-phase entry and genomic integrity. Nature.

[cit24] Jurgeit A. (2012). *et al.*, Niclosamide is a proton carrier and targets acidic endosomes with broad antiviral effects. PLoS Pathog..

[cit25] Park S. J. (2011). *et al.*, Niclosamide induces mitochondria fragmentation and promotes both apoptotic and autophagic cell death. BMB Rep..

[cit26] Weinbach E. C., Garbus J. (1969). Mechanism of action of reagents that uncouple oxidative phosphorylation. Nature.

[cit27] Figarola J. L. (2015). *et al.*, SR4 Uncouples Mitochondrial Oxidative Phosphorylation, Modulates AMP-dependent Kinase (AMPK)-Mammalian Target of Rapamycin (mTOR) Signaling, and Inhibits Proliferation of HepG2 Hepatocarcinoma Cells. J. Biol. Chem..

[cit28] Zhang P. (2022). *et al.*, Mitochondria-Related Ferroptosis Drives Cognitive Deficits in Neonatal Mice Following Sevoflurane Administration. Front. Med..

[cit29] Bains R. (2009). *et al.*, Sevoflurane and propofol depolarize mitochondria in rat and human cerebrocortical synaptosomes by different mechanisms. Acta Anaesthesiol. Scand..

[cit30] Park S. J. (2011). *et al.*, A receptor tyrosine kinase inhibitor, Tyrphostin A9 induces cancer cell death through Drp1 dependent mitochondria fragmentation. Biochem. Biophys. Res. Commun..

[cit31] Polson E. S. (2018). *et al.*, KHS101 disrupts energy metabolism in human glioblastoma cells and reduces tumor growth in mice. Sci. Transl. Med..

[cit32] Alao J. P. (2007). The regulation of cyclin D1 degradation: roles in cancer development and the potential for therapeutic invention. Mol. Cancer.

[cit33] Seftel H. C., Heinz H. J. (1964). Treatment of Human Tapeworm Infections with Yomesan: Single Dose Treatment in Non-Fasting Subjects. S. Afr. Med. J..

[cit34] PATIENT INFORMATION LEAFLET – YOMESAN CHEWABLE TABLETS, https://www.bayer.com/sites/default/files/YOMESAN_EN_PIL.pdf, (accessed April 2025)

[cit35] Li W. (2022). *et al.*, Nanchangmycin regulates FYN, PTK2, and MAPK1/3 to control the fibrotic activity of human hepatic stellate cells. Elife.

[cit36] Rausch K. (2017). *et al.*, Screening Bioactives Reveals Nanchangmycin as a Broad Spectrum Antiviral Active against Zika Virus. Cell Rep..

[cit37] Saller B. S. (2024). *et al.*, Acute suppression of mitochondrial ATP production prevents apoptosis and provides an essential signal for NLRP3 inflammasome activation. Immunity.

[cit38] Ek F. (2022). *et al.*, Sorafenib and nitazoxanide disrupt mitochondrial function and inhibit regrowth capacity in three-dimensional models of hepatocellular and colorectal carcinoma. Sci. Rep..

[cit39] Sun H. (2021). *et al.*, Nitazoxanide impairs mitophagy flux through ROS-mediated mitophagy initiation and lysosomal dysfunction in bladder cancer. Biochem. Pharmacol..

[cit40] Lee Y. (2014). *et al.*, Cyclin D1-Cdk4 controls glucose metabolism independently of cell cycle progression. Nature.

[cit41] Sakamaki T. (2006). *et al.*, Cyclin D1 determines mitochondrial function in vivo. Mol. Cell. Biol..

[cit42] Bhalla K. (2014). *et al.*, Cyclin D1 represses gluconeogenesis via inhibition of the transcriptional coactivator PGC1alpha. Diabetes.

[cit43] Van Humbeeck C. (2011). *et al.*, Parkin interacts with Ambra1 to induce mitophagy. J. Neurosci..

[cit44] Strappazzon F. (2015). *et al.*, AMBRA1 is able to induce mitophagy via LC3 binding, regardless of PARKIN and p62/SQSTM1. Cell Death Differ..

[cit45] Di Rienzo M. (2024). *et al.*, Role of AMBRA1 in mitophagy regulation: emerging evidence in aging-related diseases. Autophagy.

[cit46] Scholes N. S. , *et al.*, Inhibitor-induced supercharging of kinase turnover via endogenous proteolytic circuits, bioRxiv, 2024, preprint, 10.1101/2024.07.10.602881

