# Supplementary material for: Niclosamide: CRL4AMBRA1 mediated degradation of cyclin D1 following mitochondrial membrane depolarization
Source: RSC Med Chem. 2025 May 6;16(7):3049–57. doi: 10.1039/d5md00054h (PMC12054360; doi:10.1039/d5md00054h)
Supplement: MD-016-D5MD00054H-s001 [file MD-016-D5MD00054H-s001.pdf]

Supplementary Information (SI) for RSC Medicinal Chemistry.
This journal is © The Royal Society of Chemistry 2025

Supplementary Information

Niclosamide: $CRL4^{AMBRA1}$ mediated degradation of Cyclin D1 following mitochondrial membrane depolarization

Seemon Coomar [a], Jessica A. Gasser[b], Mikołaj Słabicki[b, c, d], Katherine A. Donovan[e, f], Eric S. Fischer[e, f], Benjamin L. Ebert[g], Dennis Gillingham[h], Nicolas H. Thomä [a, i]

[a] Friedrich Miescher Institute for Biomedical Research, Fabrikstrasse 24, Basel 4056, Switzerland
[b] Broad Institute of MIT and Harvard, Cambridge, MA 02142, USA
[c] Department of Medical Oncology, Dana-Farber Cancer Institute, Boston, MA 02115, USA
[d] Krantz Family Center for Cancer Research, Massachusetts General Hospital Cancer Center, Charlestown, MA 02129, USA
[e] Department of Cancer Biology, Dana-Farber Cancer Institute, 450 Brookline Ave., Boston, MA 02215, USA
[f] Department of Biological Chemistry and Molecular Pharmacology, Harvard Medical School, 240 Longwood Ave., Boston, MA 02215, USA
[g] Dana-Farber Cancer Institute, Boston, MA 02115, USA
[h] Department of Chemistry, University of Basel, Basel 4056, Switzerland
[i] Swiss Institute for Experimental Cancer Research (ISREC), EPFL, Station 19, Lausanne 1015, Switzerland

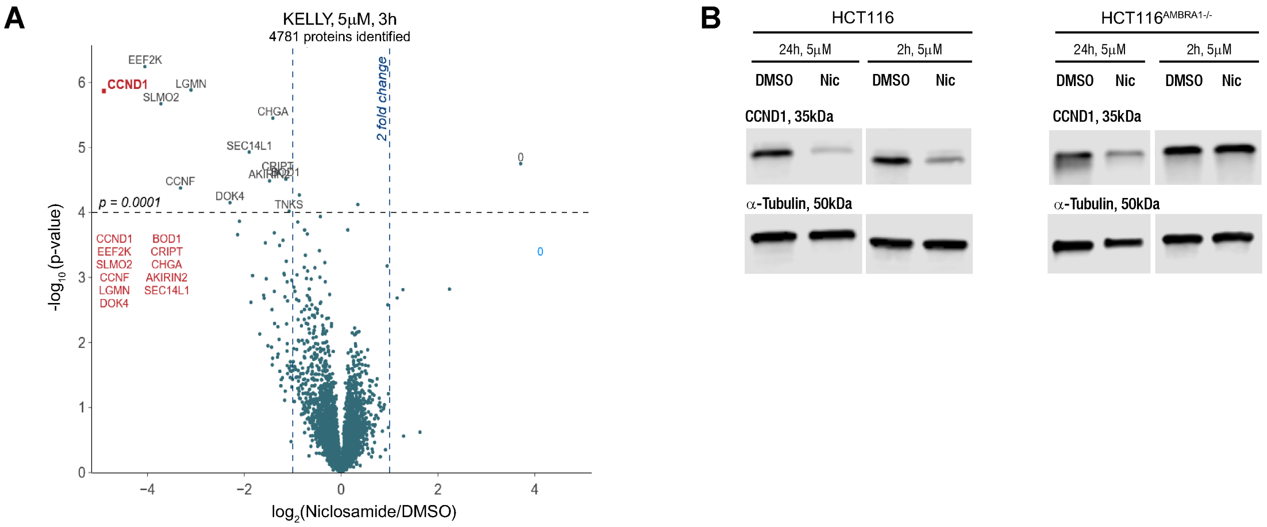

Figure S1 | Niclosamide induces AMBRA1 dependent CCND1 degradation.

A) Change in protein expression levels relative to vehicle treatment (DMSO) in KELLY cells treated with Niclosamide at 5 µM for 5 h quantified by TMT labelling and LC MS/MS analysis plotted against the p-Value. **B)** Western Blot of Anti-CCND1 and Anti-Tubulin of HCT116 and AMBRA1 KO HCT116$^{AMBRA1-/-}$ cells treated with vehicle (DMSO) or Niclosamide at 5 µM for 2h.

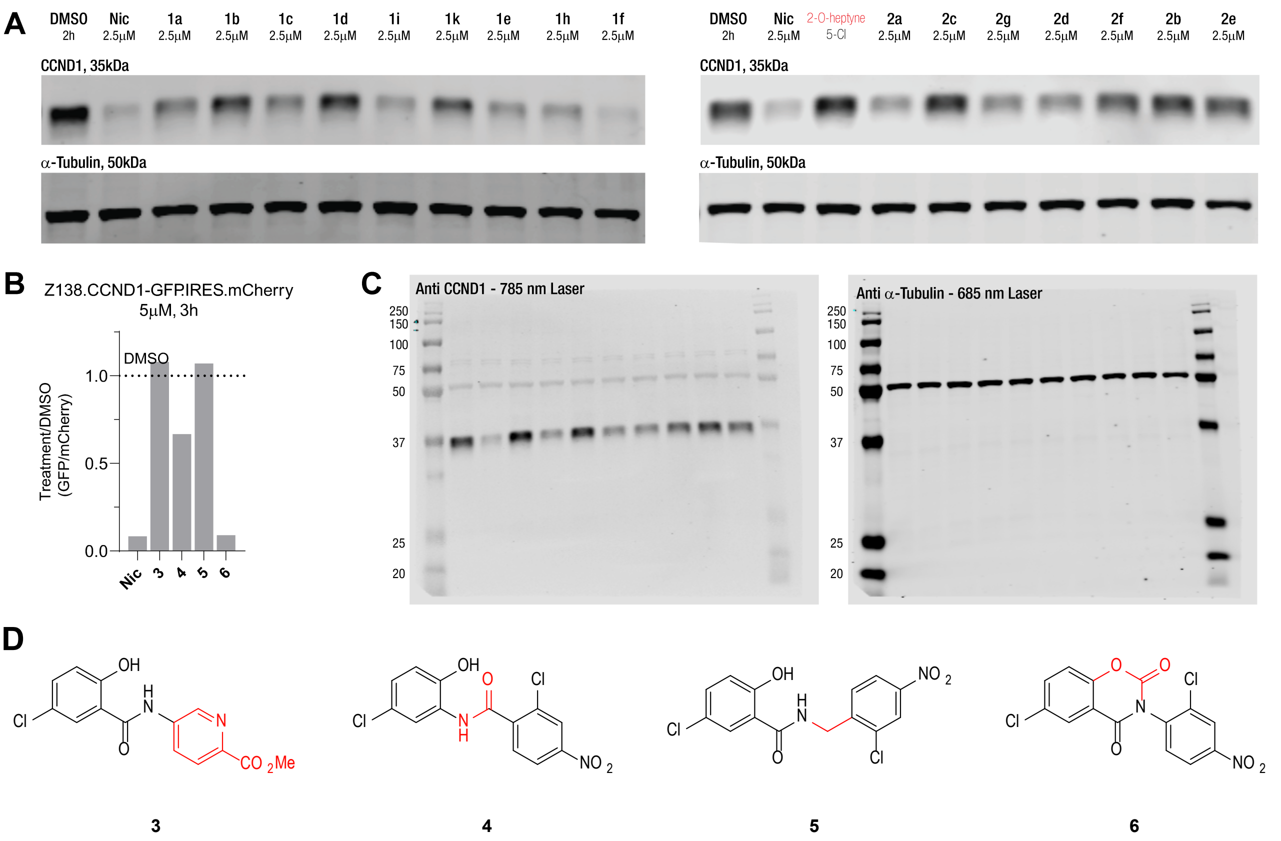

Figure S2 | Structure activity study of Niclosamide towards CCND1 degradation.

A) Western Blot of Anti-CCND1 and Anti-Tubulin of HCT116 and AMBRA1 KO HCT116$^{AMBRA1-/-}$ cells treated with vehicle (DMSO) or Niclosamide and structurally similar molecules outlined in Figure 3 at 5 µM for 2h. **B)** Change in CCND1 levels relative to vehicle (DMSO) treatment in the reporter cells treated at a concentration of 5 µM for 3h. Values represent the ratio of the geometric mean of GFP and mCherry values in the gated population. **C)** Uncropped blots of the right immunoblot shown in A) shown as a representative of the antibodies and visualizations used in the study. **D)** Molecules used for the measurements shown in B).

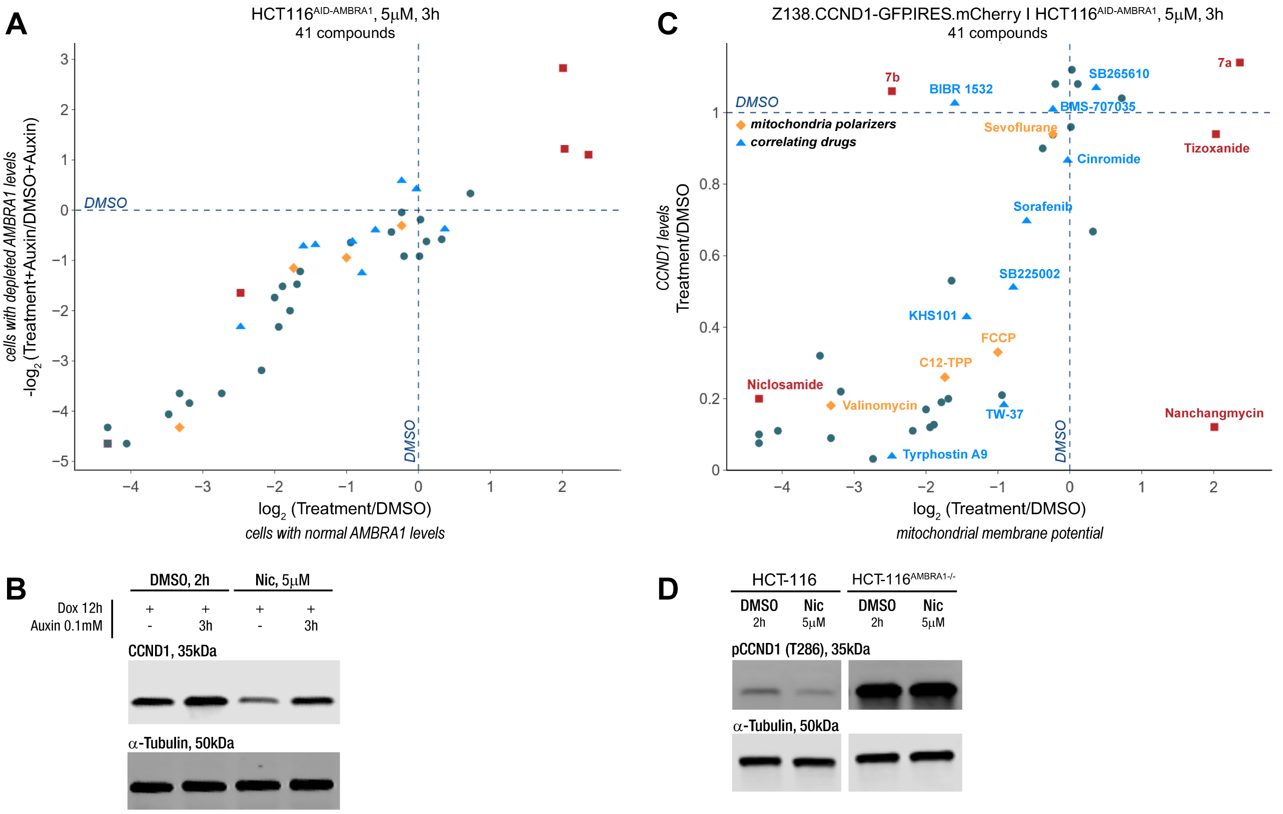

E

MACCS fingerprint

	Niclosamide	FCCP	Valinomycin	Tyrphostin A9	TW-37	KHS101	SB225002	Sorafenib
Niclosamide	1.000000	0.350000	0.344262	0.229508	0.493151	0.179487	0.807018	0.492308
FCCP	0.350000	1.000000	0.211538	0.333333	0.232877	0.216667	0.354839	0.418182
Valinomycin	0.344262	0.211538	1.000000	0.295455	0.378788	0.213115	0.328125	0.436364
Tyrphostin A9	0.229508	0.333333	0.295455	1.000000	0.292308	0.155172	0.218750	0.263158
TW-37	0.493151	0.232877	0.378788	0.292308	1.000000	0.231707	0.435897	0.376623
KHS101	0.179487	0.216667	0.213115	0.155172	0.231707	1.000000	0.187500	0.308824
SB225002	0.807018	0.354839	0.328125	0.218750	0.435897	0.187500	1.000000	0.538462
Sorafenib	0.492308	0.418182	0.436364	0.263158	0.376623	0.308824	0.538462	1.000000

Morgan fingerprint

	Niclosamide	FCCP	Valinomycin	Tyrphostin A9	TW-37	KHS101	SB225002	Sorafenib
Niclosamide	1.000000	0.139785	0.036145	0.110000	0.207143	0.133929	0.455696	0.247863
FCCP	0.139785	1.000000	0.008065	0.157303	0.110345	0.091743	0.126316	0.232143
Valinomycin	0.036145	0.008065	1.000000	0.040816	0.079310	0.038314	0.027778	0.046931
Tyrphostin A9	0.110000	0.157303	0.040816	1.000000	0.169014	0.068966	0.098039	0.091603
TW-37	0.207143	0.110345	0.079310	0.169014	1.000000	0.181818	0.214286	0.264151
KHS101	0.133929	0.091743	0.038314	0.068966	0.181818	1.000000	0.196262	0.152174
SB225002	0.455696	0.126316	0.027778	0.098039	0.214286	0.196262	1.000000	0.235294
Sorafenib	0.247863	0.232143	0.046931	0.091603	0.264151	0.152174	0.235294	1.000000

Figure S3 | AMBRA1 rescues CCND1 degradation but not mitochondrial depolarization.

A) Change in the mitochondria membrane potential (MMP) measured in HCT116$^{AID\text{-}AMBRA1}$ cells stained with JC-1 and treated with compounds discussed in Figures 4B, 5B, at 5 µM for 3h in the presence of Auxin plotted against the changes in the absence of Auxin. **B)** Western Blot of Anti-CCND1 and Anti-Tubulin of HCT116$^{AID\text{-}AMBRA1}$ cells treated with vehicle (DMSO) or Niclosamide at 5 µM for 2h with or without co-treatment of Auxin at 0.1mM for 3h. **C)** Change in CCND1 levels relative to vehicle (DMSO) quantified in reporter cells (Z138.CCND1-GFP.IRES.mCherry) treated with the compounds discussed in Figures 4B, 5B, 5D and plotted against the change in MMP measured in HCT116$^{AID\text{-}AMBRA1}$ cells treated with the same compounds at a concentration of 5 µM for 3h. **D)** Western Blot of Anti-pCCND1 (T286) and Anti-Tubulin of HCT116 and HCT116$^{AMBRA1\text{-}/\text{-}}$ cells treated with vehicle (DMSO) or Niclosamide at 5 µM for 2h. **E)** Tanimoto similarity scores were calculated using RDKit, employing both MACCS fingerprints and Morgan fingerprints (radius of 2 and vector length of 2048) to represent the molecular structures.

Biochemical experiments

Cell lines and cell culture

All unmodified wild type cell lines were purchased from ATCC, DSMZ or Takeda and used as received. During culturing they were regularly tested for mycoplasma using the Mycostrip Mycoplasma Kit (Invivogen). HCT116 cells were grown in McCoy's' 5A, Lenti-X 293T cells in DMEM, Hep3B cells in MEM, Z138 cells in IMDM and KELLY cells in RPMI. All growing media were supplemented with 10% FCS and 1% penicillin/ streptomycin. All the cells were kept in a humidified incubator at 37 °C with 5% CO_2. The engineered HCT116$^{AMBRA1-/-}$ and HCT116$^{2\times Flag\text{-}mAID\text{-}AMBRA1}$ stably infected with pTRIPZ-HA-TIR1 were kind gifts from M. Pagano, NYU Grossman School of Medicine.

Generation of Cas9 and reporter cell lines

The Hep3B.Cas9 cells, Hep3B.Cas9.CCND1-GFP.IRES.mCherry and Z138.CCND1-GFP.IRES.mCherry cell lines were generated via lentivirus transduction. The transfer plasmid for Cas9 (lentiCas9-Blast, Addgene #52962) was obtained from Addgene, whereas for the Cyclin D1 reporters the coding sequence of Cyclin D1 (P24385) was synthesized and cloned in to a Lenti virus transfer plasmid (Artichoke, Addgene #73320). To produce the viruses, 550k Lenti-X 293T cells (Takeda #632180) were seeded in a 6-well plate with 3 mL of growth medium. The following day, a plasmid solution containing psPAX2 (500 ng), pVSV-G (5 ng), and the transfer plasmid (500 ng) was diluted in 21.5 µL of OPTIMEM, reaching a total volume of 32.5 µL. A second solution consisting of 9 µL OPTIMEM and 9 µL TransIT-LT1 was prepared, incubated at room temperature for 5 minutes, and then gently mixed into the plasmid solution. The mixture was incubated at room temperature for 30 minutes before being added to the Lenti-X cells. The cells were allowed to grow for 48 hours, after which the virus-containing supernatant was centrifuged (800 g, 2 min), aliquoted in 300 µL volumes, and stored at -80 °C.

For the transduction, 3 million cells were suspended in 2.7 mL of growth medium in a six-well plate, and 300 µL of a previously thawed virus aliquot was added. The plate was centrifuged at 800 g for 2 hours at 37 °C and the cells let to grow for 48 hours in the incubator. Following this, the cells were subjected to selection with puromycin (2 µg/mL).

CRISPR screen in Hep3B cells

The CRISPR-Cas9 screen using the Bison library in Hep3B.Cas9.CCND1-GFP.IRES.mCherry reporter cells was performed as previously described [1]. The library bearing reporter cells were treated with Niclosamide or DMSO and sorted using gates as illustrated in Figure 2D, whereby each gate contained 5% of the total population in the shown window.

Data analysis of CRISPR-Cas9 knockout screens

The data analysis was performed using the pipeline described previously [1].

Cell treatments

180k cells were seeded in a 12 well-plate format in 1 mL growing medium and left to grow in the incubator for 32 h. They were then treated with compounds at the indicated concentration and for the indicated duration. The media was aspirated, the cells washed once with PBS and 0.05% Trypsin was added to detach the cells. The trypsin was neutralized by the addition of growing medium and the cells collected via centrifugation (400 g for 2 min), washed three times in PBS by resuspension and centrifugation. Finally, the PBS was removed and the cell pellets were stored at -80 °C and either used for LC-MS analysis or western blotting.

The compounds Niclosamide (Sigma Aldrich), 5-Chloro-*N*-(2-chlorophenyl)-2-hydroxybenzamide (**1b**) (Combi-Blocks), Sevoflurane (Cayman), (1-Dodecyl)triphenylphos-phonium bromide (C12-TPP) (Thermo Scientific Chemicals), BAM 15, BIBR 1532, BMS-707035, CCCP, Cinromide, COH-SR4, FCCP, KHS101, SB225002, SB-265610, Sorafenib, Tizoxanide, TW-37, Tyrphostin A9, Valinomycin (MedChem Express) were purchased and used as received.

The cells for RNA extraction and qPCR were not washed but directly collected in 1 mL TRI Reagent® (TR 118) and stored at -80 °C.

Flow cytometry experiments

For the flow cytometry experiments 120k cells were seeded in a 24 well-plate format in 0.5 mL growing medium and left to grow in the incubator for 16 h. They were then treated with compounds at the indicated concentration and for the indicated duration. The media was aspirated, the cells washed once with PBS and 0.05% Trypsin was added to detach the cells. The trypsin was neutralized by the addition of growing medium and the cells collected via centrifugation (400 g for 2 min) and resuspended in 150uL PBS. They were subsequently measured on a BD LSR II and the data analyzed with FlowJo software (version 10.5.3, TreeStar).

Western blot

The cell pellets were lysed in 50 µL lysis buffer (RIPA buffer, supplemented with Roche cOmplete™ protease inhibitor). The lysate was incubated on ice over 30 min with periodic vortexing and subsequently centrifuged at 16.9k g for 15 min at 4 °C. Protein concentrations were measured using DC Protein Assay. The lysates were incubated in loading buffer (5x, SDS, DTT, bromophenol blue, glycerol) at 98 °C for 5 min. The lysates (15 µg per lane) were separated on an 8% or 11% Tris-Glycine SDS-polyacrylamide gel and then transferred on nitrocellulose membranes (BioRad #1704158) using the BioRad Trans-Blot Turbo System. The blots were blocked using Intercept® (TBS) Blocking Buffer (LI-COR Biosciences) and probed with antibodies for Cyclin D1 (Invitrogen, MA5-16356, 1:1k dilution), Phospho-Cyclin D1 (Thr286) (CST, 3300T, 1:1k dilution) and α-Tubulin (Abcam, ab7291, 1:10k dilution). The blots were further developed using anti-Mouse (Licor, IRDye® 680RD Goat anti-Mouse IgG, 1:10k dilution) or anti-Rabbit secondary antibody (Licor, IRDye® 800CW Goat anti-Rabbit IgG, 1:10k dilution) and the bands were visualized using Licor Odyssey CLx imager. The images were processed using Empiria Studio® Software.

qRT-PCR Measurement of Gene Expression

RNA was extracted from the treated cells by using TRI Reagent® (MRC, TR118) according to the manufacturer's protocol and reverse transcribed using the standard protocol for SuperScript™ III Reverse Transcriptase (Thermo Fisher), whereby Oligo(dT)15 Primers (Promega) were used. Measurement of the gene expression was done using PowerUP SYBR Green Master Mix (Thermo Fisher) in triplicate on an Applied Biosystems StepOnePlus Instrument with GAPDH as the housekeeping gene. The data was quantified using StepOne Software v2.3 and the -ΔΔ Ct values were used for averaging across the replicates.

Primers used

GAPDH_F	*CCACTCCTCCACCTTTGAC*
GAPDH_R	*ACCCTGTTGCTGTAGCCA*
CCND1-a_F	*AGGCTGACGTGTGAGGGAGGAC*
CCND1-a_R	*AAAATGGAGCTGCGGCCTGTCC*
CCND1-b_F	*ACCCCGATGCCAACCTCCTCAA*
CCND1-b_R	*TGGACGGCAGGACCTCCTTCTG*

JC-1 assay

180k cells were seeded in a 12 well-plate format in 1 mL growing medium and left to grow in the incubator for 32 h. The media was collected and redistributed (0.5 mL per well) after which the cells were treated with compounds at 5 mM concentration for 3 h in 0.5 mL media. An aliquot of JC-1 (Invitrogen, T3168) stock (250 mM) in DMSO was added to prewarmed media to make a stock (4 mM), of which 0.5 mL was added to each well. The cells were further incubated for 15 min in the incubator after which the media got aspirated, the cells washed once with PBS and 0.05% Trypsin added to detach the cells. The trypsin was neutralized by the addition of growing medium and the cells collected via centrifugation (400 g for 2 min) and resuspended in 150 uL PBS. They were subsequently measured on a BD LSR II and the data analyzed with FlowJo software (version 10.5.3, TreeStar).

Sample preparation for TMT and PRM LC-MS/MS analysis

Cells were lysed in 50 µL lysis buffer (1% sodium deoxycholate (SDC), 0.1 M TRIS, 10 mM TCEP, pH = 8.5) using strong ultra-sonication (10 cycles, Bioruptor, Diagnode). Sample aliquots containing 60 µg of total proteins were reduced for 10 min at 95 °C and alkylated at 15 mM chloroacetamide for 30 min at 37 °C. Proteins were digested by incubation with sequencing-grade modified trypsin (1/50, w/w; Promega, Madison, Wisconsin) for 12 h at 37 °C. Tryptic digests were acidified (pH<3) using TFA

and desalted cleaned up using iST cartridges (PreOmics, Munich) according to the manufacturer's instructions. Samples were dried under vacuum and stored at -20 °C until further use.

TMT labelling

Workflow A

Sample aliquots comprising 10 µg of peptides were labeled with isobaric tandem mass tags (TMTpro 18-plex, Thermo Fisher Scientific). Peptides were resuspended in 10 µL labeling buffer (2 M urea, 0.2 M HEPES, pH 8.3) by sonication and 2.5 µL of each TMT reagent were added to the individual peptide samples followed by a 1 h incubation at 25 °C shaking at 500 rpm. To quench the labelling reaction, 0.75 µL aqueous 1.5 M hydroxylamine solution was added and samples were incubated for 5 min at 25 °C shaking at 500 rpm followed by pooling of all samples. The pH of the sample pool was increased to 11.9 by adding 1 M phosphate buffer (pH 12) and incubated for 20 min at 25 °C and 500 rpm shaking to remove TMT labels linked to peptide hydroxyl groups. Subsequently, the reaction was stopped by adding 2 M hydrochloric acid until a pH < 2 was reached. Finally, peptide samples were further acidified using 5% TFA, desalted using BioPureSPN MACRO™ SPE cartridges (Nest group) according to the manufacturer's instructions and dried under vacuum.

Workflow B

Cells were lysed by the addition of lysis buffer (8 M urea, 50 mM NaCl, 50 mM 4-(2-hydroxyethyl)-1-piperazineethanesulfonic acid (EPPS) pH 8.5, Protease and Phosphatase inhibitors) followed by manual homogenization by 20 passes through a 21-gauge (1.25 in. long) needle. Lysate was clarified by centrifugation and protein quantified using bradford (Bio-Rad) assay. 100 µg of protein for each sample was reduced, alkylated and precipitated using methanol/chloroform as previously described [2] and the resulting washed precipitated protein was allowed to air dry. Precipitated protein was resuspended in 4 M urea, 50 mM HEPES pH 7.4, buffer for solubilization, followed by dilution to 1 M urea with the addition of 200 mM EPPS, pH 8. Proteins were digested for 12 hours at room temperature with LysC (1:50 ratio), followed by dilution to 0.5 M urea and a second digestion step was performed by addition of trypsin (1:50 ratio) for 6 hours at 37 ºC. Anhydrous ACN was added to each peptide sample to a final concentration of 30%, followed by addition of Tandem mass tag (TMT) reagents at a labelling ratio of 1:4 peptide:TMT label. TMT labelling occurred over a 1.5 hour incubation at room temperature followed by quenching with the addition of hydroxylamine to a final concentration of 0.3%. Each of the samples were combined using adjusted volumes and dried down in a speed vacuum followed by desalting with C18 SPE (Sep-Pak, Waters).

TMT LC-MS/MS analysis

Workflow A

TMT-labeled peptides were fractionated by high-pH reversed phase separation using a XBridge Peptide BEH C18 column (3.5 µm, 130 Å, 1 mm x 150 mm, Waters) on an Ultimate 3000 system (Thermo Scientific). Peptides were loaded on column in buffer A and the system was run at a flow of 42 µl/min. The following gradient was used for peptide separation: from 2% B to 15% B over 3 min to 45% B over 59 min to 80% B over 3 min followed by 9 min at 80% B then back to 2% B over 1 min followed by 15 min at 2% B. Buffer A was 20 mM ammonium formate in water, pH 10 and buffer B was 20 mM ammonium formate in 90% acetonitrile, pH 10. Elution of peptides was monitored with a UV detector (205 nm, 214 nm) and a total of 36 fractions were collected, pooled into 12 fractions using a post-concatenation strategy as previously described [3] and dried under vacuum.

Dried peptides were resuspended in 0.1% aqueous formic acid and subjected to LC–MS/MS analysis using an Orbitrap Eclipse Tribrid Mass Spectrometer fitted with a Ultimate 3000 nano system, a FAIMS Pro interface (all Thermo Fisher Scientific) and a custom-made column heater set to 60 °C. Peptides were resolved using a RP-HPLC column (75 µm × 30 cm) packed in-house with C18 resin (ReproSil-Pur C18–AQ, 1.9 µm resin; Dr. Maisch GmbH) at a flow rate of 0.3 µL/min. The following gradient was used for peptide separation: from 2% B to 12% B over 5 min to 30% B over 70 min to 50% B over 15 min to 95% B over 2 min followed by 18 min at 95% B then back to 2% B over 2 min followed by 18 min at 2% B. Buffer A was 0.1% formic acid in water and buffer B was 80% acetonitrile, 0.1% formic acid in water.

The mass spectrometer was operated in DDA mode with a cycle time of 3 s. Throughout each acquisition, the FAIMS Pro interface switched between CVs of −40 V and −70 V with cycle times of 1.5 s and 1.5 s, respectively. MS1 spectra were acquired in the Orbitrap at a resolution of 120,000 and a scan range of 400 to 1600 m/z, AGC target set to "Standard" and

maximum injection time set to “Auto”. Precursors were filtered with precursor selection range set to 400–1600 m/z, monoisotopic peak determination set to “Peptide”, charge state set to 2 to 6, a dynamic exclusion of 45 s, a precursor fit of 50% in a window of 0.7 m/z and an intensity threshold of 5e3.

Precursors selected for MS2 analysis were isolated in the quadrupole with a 0.7 m/z window and collected for a maximum injection time of 35 ms with AGC target set to “Standard”. Fragmentation was performed with a CID collision energy of 30% and MS2 spectra were acquired in the IT at scan rate “Turbo”.

MS2 spectra were subjected to RTS using a human database containing 20362 entries downloaded from Uniprot on 20200417 using the following settings: enzyme was set to “Trypsin”, TMTpro16plex (K and N-term) and Carbamidomethyl (C) were set as fixed modification, Oxidation (M) was set as variable modifications, maximum missed cleavages was set to 1 and maximum variable modifications to 2. Maximum search time was set to 100 ms, the scoring threshold was set to 1.4 XCorr, 0.1 dCn, 10 ppm precursor tolerance, charge state 2 and “TMT SPS MS3 Mode” was enabled. Subsequently, spectra were filtered with a precursor selection range filter of 400–1600 m/z, precursor ion exclusion set to 25 ppm low and 25 ppm high and isobaric tag loss exclusion set to “TMTpro”. MS/MS product ions of precursors identified via RTS were isolated for an MS3 scan using the quadrupole with a 2 m/z window and ions were collected for a maximum injection time of 200 ms with a normalized AGC target set to 200%. SPS was activated and the number of SPS precursors was set to 10. Isolated fragments were fragmented with normalized HCD collision energy set to 55% and MS3 spectra were acquired in the orbitrap with a resolution of 50,000 and a scan range of 100 to 500 m/z.

Workflow B
The sample was offline fractionated into 96 fractions by high pH reverse-phase HPLC (Agilent LC1260) through an aeris peptide xb-c18 column (phenomenex) with mobile phase A containing 5% acetonitrile and 10 mM NH_4HCO_3 in LC-MS grade H_2O, and mobile phase B containing 90% acetonitrile and 5 mM NH_4HCO_3 in LC-MS grade H_2O (both pH 8.0). The resulting 96 fractions were recombined in a non-contiguous manner into 24 fractions and desalted using C18 solid phase extraction plates (SOLA, Thermo Fisher Scientific) followed by subsequent mass spectrometry analysis.

Data were collected using an Orbitrap Eclipse mass spectrometer (Thermo Fisher Scientific, San Jose, CA, USA) coupled with an UltiMate 3000 RSLCnano LC system (Thermo Fisher Scientific, San Jose, CA, USA). Peptides were separated on a 50 cm 75 µm inner diameter EasySpray ES903 microcapillary column (Thermo Fisher Scientific). Peptides were separated over a 190 min gradient of 6 - 27% acetonitrile in 1.0% formic acid with a flow rate of 300 nL/min.

Quantification was performed using a MS3-based TMT method as described previously [4]. The data were acquired using a mass range of m/z 340 – 1350, resolution 120,000, AGC target 5 x 105, maximum injection time 100 ms, dynamic exclusion of 120 seconds for the peptide measurements in the Orbitrap. Data dependent MS2 spectra were acquired in the ion trap with a normalized collision energy (NCE) set at 35%, AGC target set to 1.8 x 104 and a maximum injection time of 120 ms. MS3 scans were acquired in the Orbitrap with HCD collision energy set to 55%, AGC target set to 2 x 105, maximum injection time of 150 ms, resolution at 50,000 and with a maximum synchronous precursor selection (SPS) precursors set to 10.

TMT LC-MS/MS data analysis

Workflow A
The acquired raw-files were analysed using the SpectroMine software (v 3.2, Biognosis AG, Schlieren, Switzerland). Spectra were searched against a human database consisting of 20372 protein sequences (downloaded from Uniprot on 20220222). Standard Pulsar search settings for TMT 16 pro (“TMTpro_Quantification”) were used and resulting identifications and corresponding quantitative values were exported on the PSM level using the “Export Report” function. Acquired reporter ion intensities were employed for automated quantification and statistical analysis using the in-house developed SafeQuant R script (v2.3) [5]. This analysis included adjustment of reporter ion intensities, global data normalization by equalizing the total reporter ion intensity across all channels, data imputation using the knn algorithm, summation of reporter ion intensities per protein and channel and calculation of protein abundance ratios. To meet additional assumptions (normality and homoscedasticity) underlying the use of linear regression models and t-tests, MS-intensity signals were transformed from the linear to the log-scale. The summarized protein expression values were used for statistical testing of between condition differentially abundant

proteins. Here, empirical Bayes moderated t-tests were applied, as implemented in the R/Bioconductor limma package (http://bioconductor.org/packages/release /bioc/html/limma.html). The resulting per protein and condition comparison p-values.

Workflow B

Proteome Discoverer 2.4 (Thermo Fisher Scientific) was used for .RAW file processing and controlling peptide and protein level false discovery rates, assembling proteins from peptides, and protein quantification from peptides. The MS/MS spectra were searched against a Swissprot human database (January 2021) containing both the forward and reverse sequences. Searches were performed using a 20 ppm precursor mass tolerance, 0.6 Da fragment ion mass tolerance, tryptic peptides containing a maximum of two missed cleavages, static alkylation of cysteine (57.02146 Da), static TMT labelling of lysine residues and N-termini of peptides (229.1629 Da), and variable oxidation of methionine (15.99491 Da). TMT reporter ion intensities were measured using a 0.003 Da window around the theoretical m/z for each reporter ion in the MS3 scan. The peptide spectral matches with poor quality MS3 spectra were excluded from quantitation (summed signal-to-noise across channels < 100 and precursor isolation specificity < 0.5), and the resulting data was filtered to only include proteins with a minimum of 2 unique peptides quantified. Reporter ion intensities were normalized and scaled using in-house scripts in the R framework (R Core Team, 2014). Statistical analysis was carried out using the limma package within the R framework [6].

Chemical synthesis

N-(4-Amino-2-chloro-phenyl)-5-chloro-2-hydroxy-benzamide (1a)

As previously reported [7], Niclosamide (50 mg, 153 µmol) was dissolved in 2 mL MeOH, followed by the addition of 0.8 mL of a saturated aq. NH_4Cl solution. Subsequently, Zinc dust (100 mg, 1.53 mmol) was added to the solution on an ice bath. The mixture was stirred at rt for 16 h after which 100 mL MeOH was added. Subsequent filtration, and reduction of the filtrate *in vacuo* afforded a yellow crude which was purified using preparative RP-HPLC (Gemini NX-C18, 110 Å, 21.2*250 mm* 5 µm; mobile phase: [0.1% TFA (v/v) in H_2O, (0.1% TFA (v/v) in ACN)]) to yield **1a** (15 mg, 33%).

Data for **1a** - ^{1}H NMR (500 MHz, DMSO-d_6) δ ppm: 12.26 (s, 1H), 10.49 (s, 1H), 8.00 (d, J = 2.7 Hz, 1H), 7.82 (d, J = 8.7 Hz, 1H), 7.48 (dd, J = 8.7, 2.8 Hz, 1H), 7.03 (d, J = 8.8 Hz, 1H), 6.87 (d, J = 2.6 Hz, 1H), 6.71 (dd, J = 8.7, 2.8 Hz, 1H). ^{13}C NMR (126 MHz, DMSO-d_6) δ ppm: 163.6, 156.3, 144.0, 133.3, 129.0, 126.2, 125.7, 123.1, 119.1, 118.9, 115.4, 114.7. ESI-MS *m/z*: 298.2 $[M+H]^+$.

4-chloro-2-((2-chloro-4-nitrophenyl)carbamoyl)phenyl benzoate (2a)

As previously reported [8], to a suspension of Niclosamide (20 mg, 61 µmol) and DMAP (7.5 mg, 61 µmol) in 1 mL pyridine was added benzoyl chloride dropwise at rt. Then the mixture was stirred at 80 °C for 2 h. Subsequent dilution with 10 mL EtOAc, followed by washing with aq. HCl (1 M) and brine, followed by drying with $MgSO_4$ and concentration *in vacuo* afforded a crude which was purified by RP-HPLC (Gemini NX-C18, 110 Å, 21.2*250 mm* 5µm; mobile phase: [0.1% TFA (v/v) in H_2O, (0.1% TFA (v/v) in ACN)]) to yield **2a** (24 mg, 91%).

Data for **2a** - ^{1}H NMR (500 MHz, CD_3CN) δ ppm: 8.92 (s, 1H), 8.44 (d, J = 9.2 Hz, 1H), 8.25 (d, J = 2.6 Hz, 1H), 8.19 – 8.09 (m, 3H), 7.92 (d, J = 2.7 Hz, 1H), 7.74 – 7.64 (m, 2H), 7.57 – 7.52 (m, 1H), 7.41 (d, J = 8.7 Hz, 1H). ^{13}C NMR (126 MHz, DMSO-d_6) δ ppm: 165.8, 164.0, 148.3, 144.9, 141.3, 135.4, 133.8, 132.5, 131.2, 130.8, 130.7, 129.9, 129.6, 126.5, 125.8, 125.1, 124.3, 123.1. ESI-MS *m/z*: 432.2 $[M+H]^+$.

2-chloro-N-(2-chloro-4-nitro-phenyl)-5-hydroxy-benzamide (2b)

To a mixture of 2-chloro-5-hydroxy-benzoic acid (200 mg, 1.16 mmol), 2-chloro-4-nitro-aniline (200 mg, 1 eq) in toluene (10 mL) was added PCl_3 (65 mg, 473 µmol) dropwise at 110 °C under N_2. Then the mixture was stirred at 110 °C for 12 h. The mixture was concentrated to give the crude product. The crude product was purified by Preparative RP-HPLC (column: Phenomenex Synergi C18 150*25 mm* 10mm; mobile phase: [water (FA)-ACN]; B%: 42%-75%, 11 min). After Preparative RP-HPLC purification, the eluent was concentrated to remove organic solvents. The residual aqueous solution was lyophilized to give **2b** (153.2 mg, 39%) as a white solid.

Data for **2b** - ^{1}H NMR (500 MHz, DMSO-d_6) δ ppm: 10.51 (s, 1H), 10.07 (s, 1H), 8.39 (d, J = 2.6 Hz, 1H), 8.27 (dd, J = 2.6, 9.0 Hz, 1H), 8.13 (d, J = 9.0 Hz, 1H), 7.35 (d, J = 8.7 Hz, 1H), 7.01 (d, J = 2.9 Hz, 1H), 6.92 (dd, J = 2.9, 8.7 Hz, 1H). ^{13}C NMR (126 MHz, DMSO-d_6) δ ppm: 165.4, 156.3, 144.4, 140.8, 136.2, 130.8, 127.0, 125.9, 125.0, 123.0, 119.3, 118.5, 115.8. ESI-MS *m/z*: 324.8 ({^{35}Cl}+{^{35}Cl})$[M-H]^-$.

The following compounds were prepared by using the same strategy **1c, 1d, 1e, 1f, 1h, 1i, 1j, 1k, 1l, 1m, 1n, 2c, 2d, 2e, 2f, 2g, 2h, 2i, 2j, 7a, 7b.**

Data for **1c** - ^{1}H NMR (500 MHz, DMSO-d_6) δ ppm: 11.98 (s, 1H), 8.74 (d, J = 8.7 Hz, 1H), 8.14 (d, J = 2.9 Hz, 1H), 7.92 (d, J = 2.9 Hz, 1H), 7.85 (dd, J = 2.0, 8.7 Hz, 1H), 7.45 (dd, J = 2.8, 8.8 Hz, 1H), 7.02 (d, J = 8.8 Hz, 1H). ^{13}C NMR (126 MHz, DMSO-d_6) δ ppm: 163.2, 157.3, 140.1, 133.7, 133.0, 132.3, 129.8, 122.7, 122.2, 121.5, 119.7, 119.4, 117.8, 106.3. ESI-MS m/z: 304.8 ({^{35}Cl}+{^{35}Cl})$[M-H]^-$.

Data for **1d** - ^{1}H NMR (500 MHz, DMSO-d_6) δ ppm: 11.47 (s, 1H), 8.69 (d, J = 8.7 Hz, 1H), 8.02 (d, J = 2.0 Hz, 1H), 7.96 – 7.92 (m, 2H), 7.48 (dd, J = 2.9, 8.7 Hz, 1H), 7.06 (d, J = 8.8 Hz, 1H), 3.85 (s, 3H). ^{13}C NMR (126 MHz, DMSO-d_6) δ ppm: 164.8, 162.7, 156.1, 139.6, 133.7, 129.9, 129.9, 129.1, 125.4, 123.0, 122.4, 121.1, 119.6, 119.4, 52.3. ESI-MS m/z: 340.0 ({^{35}Cl}+{^{35}Cl})$[M-H]^+$.

Data for **1e** - ^{1}H NMR (500 MHz, DMSO-d_6) δ ppm: 11.47 (s, 1H), 8.69 (dd, J = 7.8, 9.2 Hz, 1H), 8.26 (dd, J = 2.6, 11.0 Hz, 1H), 8.18 (ddd, J = 1.2, 2.6, 9.2 Hz, 1H), 7.92 (d, J = 2.8 Hz, 1H), 7.50 (dd, J = 2.9, 8.7 Hz, 1H), 7.05 (d, J = 8.8 Hz, 1H). ^{13}C NMR (126 MHz, DMSO-d_6) δ ppm: 163.1, 156.2, 152.1, 150.1, 142.3, 133.8, 133.2, 129.7, 123.1, 121.1, 120.8, 119.4, 111.2. ESI-MS m/z: 309.0 {^{35}Cl}[M-H]$^-$.

Data for **1f** - ^{1}H NMR (500 MHz, DMSO-d_6) δ ppm: 10.96 (s, 1H), 8.44 (d, J = 1.6 Hz, 2H), 7.86 (d, J = 2.7 Hz, 1H), 7.84 (s, 1H), 7.48 (dd, J = 2.8, 8.9 Hz, 1H), 7.03 (d, J = 8.8 Hz, 1H). ^{13}C NMR (126 MHz, DMSO-d_6) δ ppm: 165.5, 156.5, 140.3, 133.2, 130.9, 130.6, 128.6, 126.5, 124.3, 122.6, 122.1, 120.3, 120.2, 119.1, 116.9. ESI-MS m/z: 383.9 {^{35}Cl}[M+H]$^+$.

Data for **1h** - ^{1}H NMR (500 MHz, DMSO-d_6) δ ppm: 11.16 (s, 0H), 8.30 – 8.23 (m, 2H), 8.02 – 7.95 (m, 2H), 7.82 (d, J = 2.8 Hz, 1H), 7.45 (dd, J = 2.8, 8.7 Hz, 1H), 7.01 (d, J = 8.8 Hz, 1H). ^{13}C NMR (126 MHz, DMSO-d_6) δ ppm: 165.1, 156.7, 144.7, 142.6, 133.0, 128.7, 124.9, 122.2, 120.8, 120.0, 119.2. ESI-MS m/z: 290.9 {^{35}Cl}[M-H]$^-$.

Data for **1i** - ^{1}H NMR (500 MHz, DMSO-d_6) δ ppm: 11.49 (s, 1H), 8.74 (dd, J = 0.9, 8.7 Hz, 1H), 7.97 (d, J = 1.3 Hz, 1H), 7.96 (d, J = 2.8 Hz, 1H), 7.77 (ddd, J = 0.8, 2.3, 8.7 Hz, 1H), 7.49 (dd, J = 2.9, 8.7 Hz, 1H), 7.07 (d, J = 8.8 Hz, 1H). ^{13}C NMR (126 MHz, DMSO-d_6) δ ppm: 162.9, 156.2, 139.0, 133.7, 129.9, 126.4, 125.2, 124.8, 124.6, 123.0, 122.4, 121.9, 119.5, 119.4. ESI-MS m/z: 347.8 ({^{35}Cl}+{^{35}Cl})[M-H]$^-$.

Data for **1j** - ^{1}H NMR (500 MHz, DMSO-d_6) δ ppm: 10.57 (s, 1H), 7.97 (d, J = 2.7 Hz, 1H), 7.69 (t, J = 2.0 Hz, 1H), 7.57 (d, J = 9.2 Hz, 1H), 7.44 (dd, J = 2.7, 8.8 Hz, 1H), 7.29 (t, J = 7.9 Hz, 1H), 7.18 (d, J = 8.7 Hz, 1H), 6.99 (d, J = 8.8 Hz, 1H), 1.29 (s, 9H). ^{13}C NMR (126 MHz, DMSO-d_6) δ ppm: 165.2, 157.7, 151.4, 137.8, 133.0, 128.4, 128.2, 122.2, 121.2, 119.4, 119.3, 118.1, 117.8, 34.5, 31.1.ESI-MS m/z: 302.1 {^{35}Cl}[M-H]$^-$.

Data for **1k** - ^{1}H NMR (500 MHz, DMSO-d_6) δ ppm: 11.02 (s, 1H), 8.34 (dd, J = 5.9, 9.2 Hz, 1H), 7.97 (d, J = 2.8 Hz, 1H), 7.58 (dd, J = 2.9, 8.5 Hz, 1H), 7.48 (dd, J = 2.8, 8.8 Hz, 1H), 7.29 (ddd, J = 3.0, 8.2, 9.2 Hz, 1H), 7.05 (d, J = 8.8 Hz, 1H). ^{13}C NMR (126 MHz, DMSO-d_6) δ ppm: 163.2, 159.3, 157.3, 156.4, 133.4, 131.9, 129.5, 125.0, 124.6, 122.9, 119.3, 116.3, 114.5. ESI-MS m/z: 297.8 ({^{35}Cl}+{^{35}Cl})[M-H]$^-$.

Data for **1l** - ^{1}H NMR (500 MHz, DMSO-d_6) δ ppm: 11.18 (s, 1H), 8.45 (d, J = 8.8 Hz, 1H), 7.95 (d, J = 2.8 Hz, 1H), 7.73 (d, J = 2.4 Hz, 1H), 7.48 (dt, J = 2.4, 8.9 Hz, 2H), 7.06 (d, J = 8.8 Hz, 1H). ^{13}C NMR (126 MHz, DMSO-d_6) δ ppm: 162.8, 156.2, 134.4, 133.5, 129.7, 128.8, 128.1, 127.9, 124.2, 123.5, 123.0, 119.5, 119.3. ESI-MS m/z: 313.8 ({35Cl}*3)[M-H]- ; 315.8 ({^{35}Cl}*2+{^{37}Cl})[M-H]$^-$.

Data for **1m** - ^{1}H NMR (500 MHz, DMSO-d_6) δ ppm: 12.17 (s, 1H), 8.56 (d, J = 9.0 Hz, 1H), 8.19 (d, J = 2.5 Hz, 1H), 7.91 (d, J = 2.8 Hz, 1H), 7.86 (dd, J = 2.6, 9.0 Hz, 1H), 7.48 (dd, J = 2.8, 8.8 Hz, 1H), 7.03 (d, J = 8.8 Hz, 1H). ^{13}C NMR (126 MHz, DMSO-d_6) δ ppm: 163.6, 156.4, 139.7, 134.5, 133.7, 132.1, 129.8, 127.6, 125.9, 125.0, 122.8, 119.6, 119.3. ESI-MS m/z: 324.8 ({^{35}Cl}+{^{35}Cl})[M-H]$^-$.

Data for **1n** - ^{1}H NMR (500 MHz, DMSO-d_6) δ ppm: 11.09 (s, 1H), 8.64 (t, J = 2.0 Hz, 1H), 8.24 (t, J = 1.9 Hz, 1H), 8.01 (t, J = 2.0 Hz, 1H), 7.82 (d, J = 2.7 Hz, 1H), 7.46 (dd, J = 2.7, 8.8 Hz, 1H), 7.01 (d, J = 8.8 Hz, 1H). ^{13}C NMR (126 MHz, DMSO-d_6) δ ppm: 165.9, 157.3, 149.1, 141.1, 134.5, 133.6, 129.1, 125.8, 122.8, 120.8, 119.7, 118.6, 113.9.ESI-MS m/z: 324.8 ({^{35}Cl}+{^{35}Cl})[M-H]$^-$.

Data for **2c** - ^{1}H NMR (500 MHz, DMSO-d_6) δ ppm: 8.39 (d, J = 2.6 Hz, 1H), 8.24 (dd, J = 2.6, 9.0 Hz, 1H), 7.98 (d, J = 9.0 Hz, 1H), 7.78 (d, J = 2.4 Hz, 1H), 7.28 (dd, J = 2.5, 8.9 Hz, 1H), 6.82 (d, J = 8.8 Hz, 1H). ^{13}C NMR (126 MHz, DMSO-d_6) δ ppm: 167.2, 149.2, 144.2, 141.8, 132.7, 128.3, 128.2, 126.9, 124.9, 122.8, 118.6, 118.0, 114.2. ESI-MS m/z: 323.8 ({^{35}Cl}+{^{35}Cl})[M-H]$^-$.

Data for **2d** - ^{1}H NMR (500 MHz, DMSO-d_6) δ ppm: 12.38 (s, 1H), 8.86 (d, J = 9.3 Hz, 1H), 8.40 (d, J = 2.7 Hz, 1H), 8.27 (dd, J = 2.7, 9.2 Hz, 1H), 7.98 (d, J = 8.6 Hz, 1H), 6.98 (d, J = 2.1 Hz, 1H), 6.92 (dd, J = 2.1, 8.6 Hz, 1H). ^{13}C NMR (126 MHz,

DMSO-d_6) δ ppm: 163.9, 160.3, 142.0, 138.1, 132.5, 124.8, 123.8, 122.2, 120.6, 118.4, 117.5, 117.1. ESI-MS m/z: 324.8 ({^{35}Cl}+{^{35}Cl})[M-H]$^-$.

Data for **2e** - ^{1}H NMR (500 MHz, DMSO-d_6) δ ppm: 10.28 (s, 1H), 8.39 (d, *J* = 2.6 Hz, 1H), 8.26 (dd, *J* = 2.6, 9.0 Hz, 1H), 8.19 (d, *J* = 9.0 Hz, 1H), 7.55 (d, *J* = 8.5 Hz, 1H), 6.92 (d, *J* = 2.3 Hz, 1H), 6.85 (dd, *J* = 2.3, 8.5 Hz, 1H). ^{13}C NMR (126 MHz, DMSO-d_6) δ ppm: 165.2, 160.1, 144.0, 141.1, 131.5, 131.4, 126.5, 125.7, 125.2, 125.0, 123.1, 116.5, 114.2. ESI-MS m/z: 326.9 ({^{35}Cl}+{^{35}Cl})[M-H]$^+$.

Data for **2f** - ^{1}H NMR (500 MHz, DMSO-d_6) δ ppm: 10.55 (s, 1H), 8.37 (d, J = 2.9 Hz, 1H), 8.33 – 8.22 (m, 2H), 7.27 (t, *J* = 8.2 Hz, 1H), 6.95 (d, *J* = 7.7 Hz, 1H), 6.90 (d, *J* = 8.2 Hz, 1H). ^{13}C NMR (126 MHz, DMSO-d_6) δ ppm: 164.2, 155.8, 143.9, 141.0, 131.0, 130.7, 125.8, 125.6, 125.2, 124.6, 123.3, 119.3, 114.6. ESI-MS m/z: 324.8 ({^{35}Cl}+{^{35}Cl})[M-H]$^-$.

Data for **2g** - ^{1}H NMR (500 MHz, DMSO-d_6) δ ppm: 11.85 (s, 1H), 8.84 (d, *J* = 9.3 Hz, 1H), 8.42 (d, *J* = 2.7 Hz, 1H), 8.28 (dd, *J* = 2.7, 9.3 Hz, 1H), 7.71 (dd, *J* = 3.3, 9.7 Hz, 1H), 7.35 (ddd, *J* = 3.4, 7.7, 9.0 Hz, 1H), 7.06 (dd, *J* = 4.6, 9.0 Hz, 1H). ^{13}C NMR (126 MHz, DMSO-d_6) δ ppm: 163.2, 156.2, 154.3, 153.6, 142.3, 141.7, 124.8, 123.9, 122.4, 121.5, 120.6, 118.9, 115.9. ESI-MS m/z: 308.8 {^{35}Cl}[M-H]$^-$.

Data for **2h** - ^{1}H NMR (500 MHz, DMSO-d_6) δ ppm: 10.74 (s, 1H), 8.39 (d, *J* = 2.7 Hz, 1H), 8.28 (dd, *J* = 2.6, 9.0 Hz, 1H), 8.18 (d, *J* = 9.0 Hz, 1H), 7.76 – 7.73 (m, 2H), 7.52 (dd, *J* = 2.7, 8.6 Hz, 1H). ^{13}C NMR (126 MHz, DMSO-d_6) δ ppm: 165.4, 144.1, 141.5, 140.0, 134.5, 132.3, 131.2, 128.9, 126.8, 125.7, 125.0, 123.0, 117.6. ESI-MS m/z: 386.7 ({^{35}Cl}*2+{^{79}Br})[M-H]$^-$; 388.7 ({^{35}Cl}+{^{37}Cl}+{^{79}Br})}) [M-H]$^-$ & ({^{35}Cl}*2+{^{81}Br})[M-H]$^-$.

Data for **2i** - ^{1}H NMR (500 MHz, DMSO-d_6) δ ppm: 10.52 (s, 1H), 8.41 (d, *J* = 2.6 Hz, 1H), 8.26 (dd, *J* = 2.6, 8.9 Hz, 1H), 8.07 – 7.99 (m, 2H), 7.96 (d, *J* = 7.8 Hz, 1H), 7.75 – 7.69 (m, 1H), 7.60 (t, *J* = 7.9 Hz, 1H). ^{13}C NMR (126 MHz, DMSO-d_6) δ ppm: 164.4, 144.6, 141.6, 135.7, 133.3, 132.0, 130.6, 128.5, 127.7, 127.1, 126.7, 124.9, 122.9. ESI-MS m/z: 308.8 ({^{35}Cl}+{^{35}Cl})[M-H]$^-$.

Data for **2j** - ^{1}H NMR (500 MHz, DMSO-d_6) δ ppm: 12.19 (s, 1H), 11.46 (s, 1H), 8.85 (d, *J* = 9.3 Hz, 1H), 8.41 (d, *J* = 2.7 Hz, 1H), 8.28 (dd, *J* = 2.6, 9.2 Hz, 1H), 8.04 (dd, *J* = 1.8, 7.9 Hz, 1H), 7.48 (ddd, *J* = 1.8, 7.1, 8.8 Hz, 1H), 7.07 (d, *J* = 8.2 Hz, 1H), 7.02 (t, *J* = 7.6 Hz, 1H). ^{13}C NMR (126 MHz, DMSO-d_6) δ ppm: 163.9, 156.4, 142.3, 141.6, 134.5, 131.2, 124.8, 123.9, 122.2, 120.6, 120.0, 117.9, 117.1. ESI-MS m/z: 291.0 {^{35}Cl}[M-H]$^-$.

Data for **7a** - ^{1}H NMR (500 MHz, DMSO-d_6) δ ppm: 12.44 (s, 1H), 7.92 (dd, *J* = 1.8, 7.9 Hz, 1H), 7.54 (s, 1H), 7.43 (ddd, *J* = 1.8, 7.2, 8.3 Hz, 1H), 7.01 – 6.91 (m, 2H). ^{13}C NMR (126 MHz, DMSO-d_6) δ ppm: 165.3, 158.0, 157.3, 135.0, 134.1, 130.1, 119.2, 117.8, 117.1. ESI-MS m/z: 255.0 {^{35}Cl}[M+H]$^+$.

Data for **7b** - ^{1}H NMR (500 MHz, DMSO-d_6) δ ppm: 8.61 (s, 1H), 8.13 (s, 1H), 7.83 (d, *J* = 2.8 Hz, 1H), 7.43 (dd, *J* = 2.8, 8.8 Hz, 1H), 6.96 (d, *J* = 8.7 Hz, 1H). ^{13}C NMR (126 MHz, DMSO-d_6) δ ppm: 163.5, 157.2, 144.1, 140.0, 133.8, 129.4, 122.7, 120.2, 119.6. ESI-MS m/z: 297.8 {^{35}Cl}[M-H]$^-$.

5-chloro-2-methoxy-benzoyl chloride

To a solution of 5-chloro-2-methoxy-benzoic acid (1 g, 5.36 mmol) in DCM (10 mL) was added $SOCl_2$ (765 mg, 6.43 mmol) dropwise at 0 °C under N_2. A catalytic amount of DMF (10 mg, 136 µmol) was added and the mixture was stirred at 30 °C for 5 h. TLC (Petroluem/ EtOAc=1:1, MeOH quenched) showed 5-chloro-2-methoxy-benzoic acid was consumed completely and a new spot was detected. The mixture was concentrated to give 5-chloro-2-methoxy-benzoyl chloride (1.1 g, crude) as a white solid.

5-chloro-*N*-(2-chloro-4-sulfamoyl-phenyl) -2-methoxy-benzamide

A mixture of 5-chloro-2-methoxy-benzoyl chloride (200 mg, 975.43 µmol), 4-amino-3-chloro-benzenesulfonamide (200 mg, 967.82 µmol) in DCM (2 mL) was stirred at 30 °C for 12 h. The mixture was concentrated to give the crude product. The crude product was diluted in sat. $NaHCO_3$ (aq, 30 mL) and EtOAc (30 mL). The mixture was filtered and the cake was dried under vacuum to give 5-chloro-N-(2-chloro-4-sulfamoyl-phenyl) -2-methoxy-benzamide (200 mg, 55%) as a white solid.

Data for **5-chloro-*N*-(2-chloro-4-sulfamoyl-phenyl) -2-methoxy-benzamide** - ^{1}H NMR (400 MHz, DMSO-d_6) δ ppm: 8.60 (d, J = 8.4 Hz, 1H), 7.98 (d, J = 2.8 Hz, 1H), 7.95 (d, J = 2.0 Hz, 1H), 7.81 (dd, J = 2.0, 8.8 Hz, 1H), 7.69 (dd, J = 2.8, 8.8 Hz, 1H), 7.36 (d, J = 8.8 Hz, 1H), 4.09 (s, 3H).

5-chloro-*N*-(2-chloro-4-sulfamoyl-phenyl) -2-hydroxy-benzamide (1g)

To a mixture of 5-chloro-*N*-(2-chloro-4-sulfamoyl-phenyl) -2-methoxy-benzamide (200 mg, 533.01 μmol, 1 eq) in DCM (10 mL) was added BBr_3 (260 mg, 1.04 mmol, 1.95 eq), then the mixture was stirred at 30 °C for 12 h. The mixture was quenched with MeOH (10 mL) and concentrated to give crude product. The crude product was purified by preparative RP-HPLC (column: Phenomenex C18 75*30 mm*3μm; mobile phase: [water (FA) -ACN]; B%: 30%-60%, 7 min). After Preparative RP-HPLC purification, the eluent was concentrated to remove organic solvents. The residual aqueous solution was lyophilized to give **1g** (102.3 mg, 52% yield) as a white solid.

Data for **1g** - ^{1}H NMR (500 MHz, DMSO-d_6) δ ppm: 11.66 (s, 1H), 8.70 (d, J = 8.8 Hz, 1H), 7.95 (d, J = 2.9 Hz, 1H), 7.94 (d, J = 2.2 Hz, 1H), 7.81 (dd, J = 2.2, 8.7 Hz, 1H), 7.47 (dd, J = 2.9, 8.8 Hz, 1H), 7.45 (s, 2H), 7.05 (d, J = 8.8 Hz, 1H). ^{13}C NMR (126 MHz, DMSO-d_6) δ ppm: 163.0, 156.9, 139.7, 138.3, 133.6, 129.8, 126.7, 125.5, 122.6, 121.7, 119.6, 119.5. ESI-MS m/z: 361.0 ({^{35}Cl}+{^{35}Cl}) [M+H]$^{+}$.

methyl 5-[(5-chloro-2-hydroxy-benzoyl)amino]pyridine-2-carboxylate (3)

To a mixture of 5-chloro-2-hydroxy-benzoic acid (200 mg, 1.16 mmol), methyl 5-aminopyridine-2-carboxylate (180 mg, 1.18 mmol) in DMF (2 mL) was added HATU (500 mg, 1.31 mmol) and DIEA (449 mg, 3.48 mmol), then the mixture was stirred at 50 °C for 12 h. The mixture was filtered and the mixture was concentrated to give the crude product. The crude product was purified by preparative RP-HPLC (column: Waters Xbridge 150*25 mm* 5um; mobile phase: [water (NH_4HCO_3) -ACN]; B%: 12%-42%, 10min). After Preparative RP-HPLC purification, the eluent was concentrated to remove organic solvents. The residual aqueous solution was lyophilized to give **3** (25.3 mg, 7%) as a white solid.

Data for **3** - ^{1}H NMR (500 MHz, DMSO-d_6) δ ppm: 11.46 (s, 1H), 8.95 (dd, J = 0.7, 2.6 Hz, 1H), 8.38 (dd, J = 2.5, 8.6 Hz, 1H), 8.09 (dd, J = 0.7, 8.6 Hz, 1H), 7.84 (d, J = 2.7 Hz, 1H), 7.43 (dd, J = 2.7, 8.8 Hz, 1H), 6.98 (d, J = 8.8 Hz, 1H), 3.87 (s, 3H). ^{13}C NMR (126 MHz, DMSO-d_6) δ ppm: 165.4, 164.7, 157.8, 141.9, 141.4, 138.4, 133.1, 128.7, 126.9, 125.6, 121.6, 120.1, 119.5, 52.2. ESI-MS m/z: 306.8 {^{35}Cl}[M+H]$^{+}$.

tert-butyl *N*-[(2-chloro-4-nitro-phenyl)methyl]carbamate

To a mixture of 2-chloro-4-nitro-benzonitrile (300 mg, 1.64 mmol, 1 eq) in THF (10 mL) was added BH_3-Me_2S (10 M, 0.2 mL, 1.22 eq) dropwise at 0 °C under N_2, then the mixture was stirred at 60 °C for 12 h. MeOH (2 mL), TEA (350 mg, 3.46 mmol, 2.10 eq) and Boc_2O (500 mg, 2.29 mmol, 1.39 eq) was added dropwise in turns, then the mixture was stirred at 20°C for 12 h. The mixture was concentrated to give crude product. The crude product was purified by flash silica gel chromatography (ISCO®; 20 g SepaFlash® Silica Flash Column, Eluent of 0~20% Ethyl acetate/Petroleum ethergradient @ 20 mL/min) to give *tert*-butyl *N*-[(2-chloro-4-nitro-phenyl) methyl]carbamate (300 mg, 1.05 mmol, 64%) as colorless oil.

Data for **tert-butyl N-[(2-chloro-4-nitro-phenyl)methyl]carbamate** - ^{1}H NMR (400 MHz, DMSO-d_6) δ ppm: 8.25 (d, J = 2.0 Hz, 1H), 8.13 (dd, J = 2.4, 8.8 Hz, 1H), 7.59 (d, J = 8.8 Hz, 1H), 5.09 (s, 1H), 4.48 (br d, J = 6.4 Hz, 2H), 1.47 (s, 9H).

(2-chloro-4-nitro-phenyl)methanamine

To a mixture of *tert*-butyl *N*-[(2-chloro-4-nitro-phenyl) methyl]carbamate (300 mg, 1.05 mmol, 1 eq) in EtOAc (10 mL) was added HCl/dioxane (4 M, 1.20 mL, 4.59 eq), then the mixture was stirred at 20 °C for 12 h. The mixture was concentrated to give (2-chloro-4-nitro-phenyl) methanamine (200 mg, crude) as a white solid.

2-chloro-*N*-(5-chloro-2-hydroxyphenyl)-4-nitrobenzamide (4)

To a suspension of 2-chloro-4-nitro-benzoyl chloride (50 mg, 23 μmol) and DIPEA (25 μL, 42 μmol) in 1 mL DCM was added 2-amino-4-chloro-phenol (30 mg, 20 μmol). The mixture was stirred at rt for 12 h. Subsequent dilution with 10 mL EtOAc, washing with brine, followed by drying with $MgSO_4$ and concentration *in vacuo* afforded a crude which was purified by RP-

HPLC (Gemini NX-C18, 110 Å, 21.2*250 mm* 5µm; mobile phase: [0.1% TFA (v/v) in H_2O, (0.1% TFA (v/v) in ACN)]) to yield **4** (25 mg, 37%).

Data for **4** - ^{1}H NMR (500 MHz, CD_3CN) δ ppm: 10.13 (s, 1H), 8.37 (d, J = 2.2 Hz, 1H), 8.27 (dd, J = 2.2, 8.4 Hz, 1H), 8.01 (d, J = 2.6 Hz, 1H), 7.85 (d, J = 8.4 Hz, 1H), 7.07 (dd, J = 2.7, 8.7 Hz, 1H), 6.92 (d, J = 8.7 Hz, 1H). ^{13}C NMR (126 MHz, DMSO-d_6) δ ppm: 163.9, 148.3, 147.6, 142.2, 131.2, 130.2, 126.6, 125.0, 124.4, 122.3, 122.0, 116.7. ESI-MS *m/z*: 432.2 [M+H]$^+$.

5-chloro-*N*-[(2-chloro-4-nitro-phenyl)methyl]-2-hydroxy-benzamide (5)

To a mixture of 5-chloro-2-hydroxy-benzoic acid (100 mg, 579 µmol, 1 eq), (2-chloro-4-nitro-phenyl) methanamine (150 mg, 672 µmol, 1.16 eq, HCl salt) in MeCN (2 mL) was added [chloro(dimethylamino) methylene]-dimethyl-ammonium; hexafluorophosphate (200 mg, 712 µmol, 1.23 eq) and 1-methylimidazole (150 mg, 1.83 mmol, 3.15 eq), then the mixture was stirred at 20 °C for 2 h. The mixture was poured into water (30 mL) and the mixture was extracted with EtOAc (30 mL*3). The organic layers were dried over Na_2SO_4, filtered and concentrated to give crude product. The crude product was purified by preparative RP-HPLC (column: Phenomenex C18 75*30 mm*3µm;mobile phase: [water (FA) -ACN]; B%: 48%-78%, 7 min). After Preparative RP-HPLC purification, the eluent was concentrated to remove organic solvents. The residual aqueous solution was lyophilized to give the product. The product was purified by preparative RP-HPLC (column: Waters Xbridge 150*25mm*5µm;mobile phase: [water (NH_4HCO_3) -ACN]; B%: 39%-69%, 9 min). After Preparative RP-HPLC purification, the eluent was concentrated to remove organic solvents. The residual aqueous solution was lyophilized to give **5** (63.2 mg, 185.26 µmol, 32% yield) as a white solid.

Data for **5** - ^{1}H NMR (500 MHz, DMSO-d_6) δ ppm: 12.16 (s, 1H), 9.48 (t, J = 5.8 Hz, 1H), 8.31 (d, J = 2.3 Hz, 1H), 8.19 (dd, J = 2.3, 8.6 Hz, 1H), 7.96 (d, J = 2.7 Hz, 1H), 7.65 (d, J = 8.6 Hz, 1H), 7.46 (dd, J = 2.7, 8.8 Hz, 1H), 6.99 (d, J = 8.8 Hz, 1H), 4.66 (d, J = 5.1 Hz, 2H). ^{13}C NMR (126 MHz, DMSO-d_6) δ ppm: 167.2, 158.0, 147.1, 143.6, 133.4, 132.6, 129.5, 128.0, 124.1, 122.5, 122.3, 119.3, 117.4. ESI-MS m/z : 341.1 ({^{35}Cl}+{^{35}Cl}) [M+H]$^+$.

6-chloro-3-(2-chloro-4-nitro-phenyl)-1,3-benzoxazine-2,4-dione (6)

To a solution of 5-chloro-*N*-(2-chloro-4-nitro-phenyl) -2-hydroxy-benzamide (200 mg, 611.40 µmol, 1 eq), TEA (200 mg, 1.98 mmol, 3.23 eq) in THF (2 mL) was added Triphosgene (200 mg, 673.97 µmol, 1.10 eq), then the mixture was stirred at 20 °C for 12 h. The mixture was concentrated to give crude product. The product was purified by flash silica gel chromatography (ISCO®; 20 g SepaFlash® Silica Flash Column, Eluent of 0~30% THF/Petroleum ether gradient @20 mL/min) to give **6** (62.3 mg, 176.43 µmol, 29% yield) as a white solid.

Data for **6** - ^{1}H NMR (500 MHz, DMSO-d_6) δ ppm: 8.58 (d, J = 2.4 Hz, 1H), 8.43 (dd, J = 2.5, 8.7 Hz, 1H), 8.05 (d, J = 2.6 Hz, 1H), 8.01 (dd, J = 2.7, 8.8 Hz, 1H), 7.98 (d, J = 8.7 Hz, 1H), 7.68 (d, J = 8.8 Hz, 1H). ^{13}C NMR (126 MHz, DMSO-d_6) δ ppm: 158.7, 151.3, 148.6, 145.9, 137.8, 137.0, 133.1, 132.2, 130.0, 126.6, 125.1, 123.6, 119.2, 115.4.

Bibliography

1. Zou, C., et al., *The human E3 ligase RNF185 is a regulator of the SARS-CoV-2 envelope protein.* iScience, 2023. **26**(5): p. 106601.
2. Donovan, K.A., et al., *Thalidomide promotes degradation of SALL4, a transcription factor implicated in Duane Radial Ray syndrome.* Elife, 2018. **7**.
3. Wang, Y., et al., *Reversed-phase chromatography with multiple fraction concatenation strategy for proteome profiling of human MCF10A cells.* Proteomics, 2011. **11**(10): p. 2019-26.
4. McAlister, G.C., et al., *MultiNotch MS3 enables accurate, sensitive, and multiplexed detection of differential expression across cancer cell line proteomes.* Anal Chem, 2014. **86**(14): p. 7150-8.
5. Ahrne, E., et al., *Evaluation and Improvement of Quantification Accuracy in Isobaric Mass Tag-Based Protein Quantification Experiments.* J Proteome Res, 2016. **15**(8): p. 2537-47.
6. Ritchie, M.E., et al., *limma powers differential expression analyses for RNA-sequencing and microarray studies.* Nucleic Acids Res, 2015. **43**(7): p. e47.
7. Zhou, J.C., Haijun; Shen, Qiang, *WO2014113467 - STAT3 INHIBITOR*. 2014.
8. Fomovska, A., et al., *Salicylanilide inhibitors of Toxoplasma gondii.* J Med Chem, 2012. **55**(19): p. 8375-91.
