# Supplementary material for: Niclosamide: CRL4AMBRA1 mediated degradation of cyclin D1 following mitochondrial membrane depolarization
Source: RSC Med Chem. 2025 May 6. Online ahead of print. doi: 10.1039/d5md00054h (PMC12054360; doi:10.1039/d5md00054h)
Supplement: Supplementary file 1 [file MD-OLF-D5MD00054H-s002.pdf]

Supplementary Information (SI) for RSC Medicinal Chemistry.
This journal is © The Royal Society of Chemistry 2025

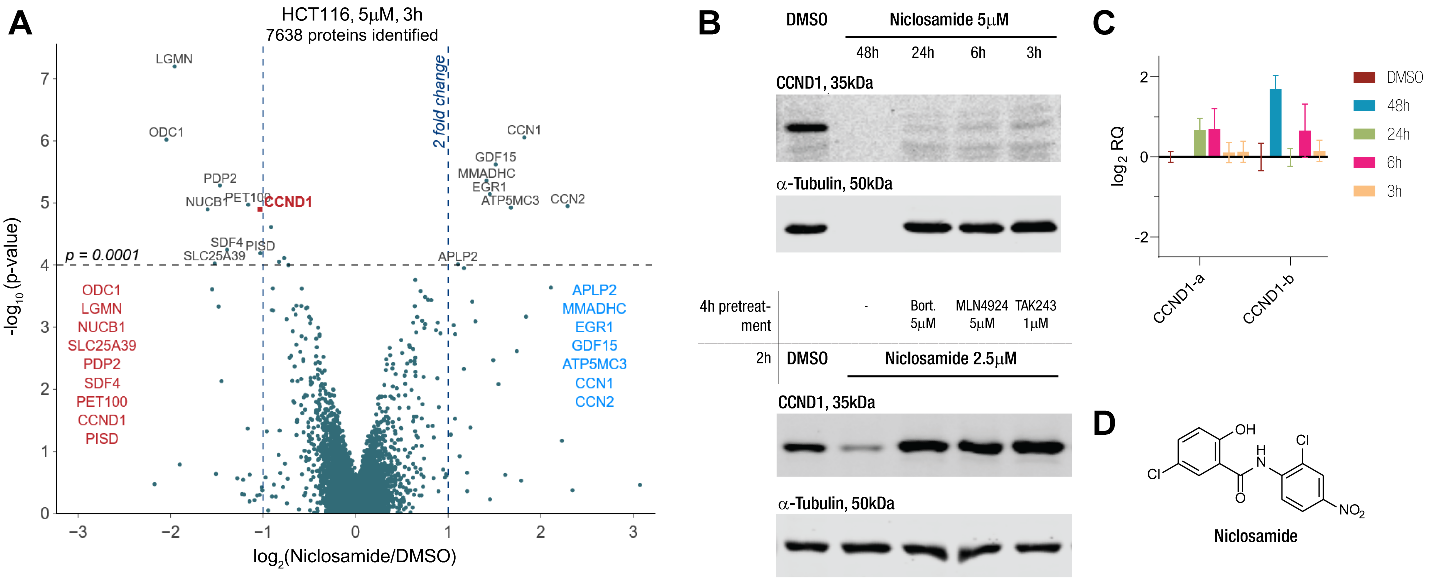

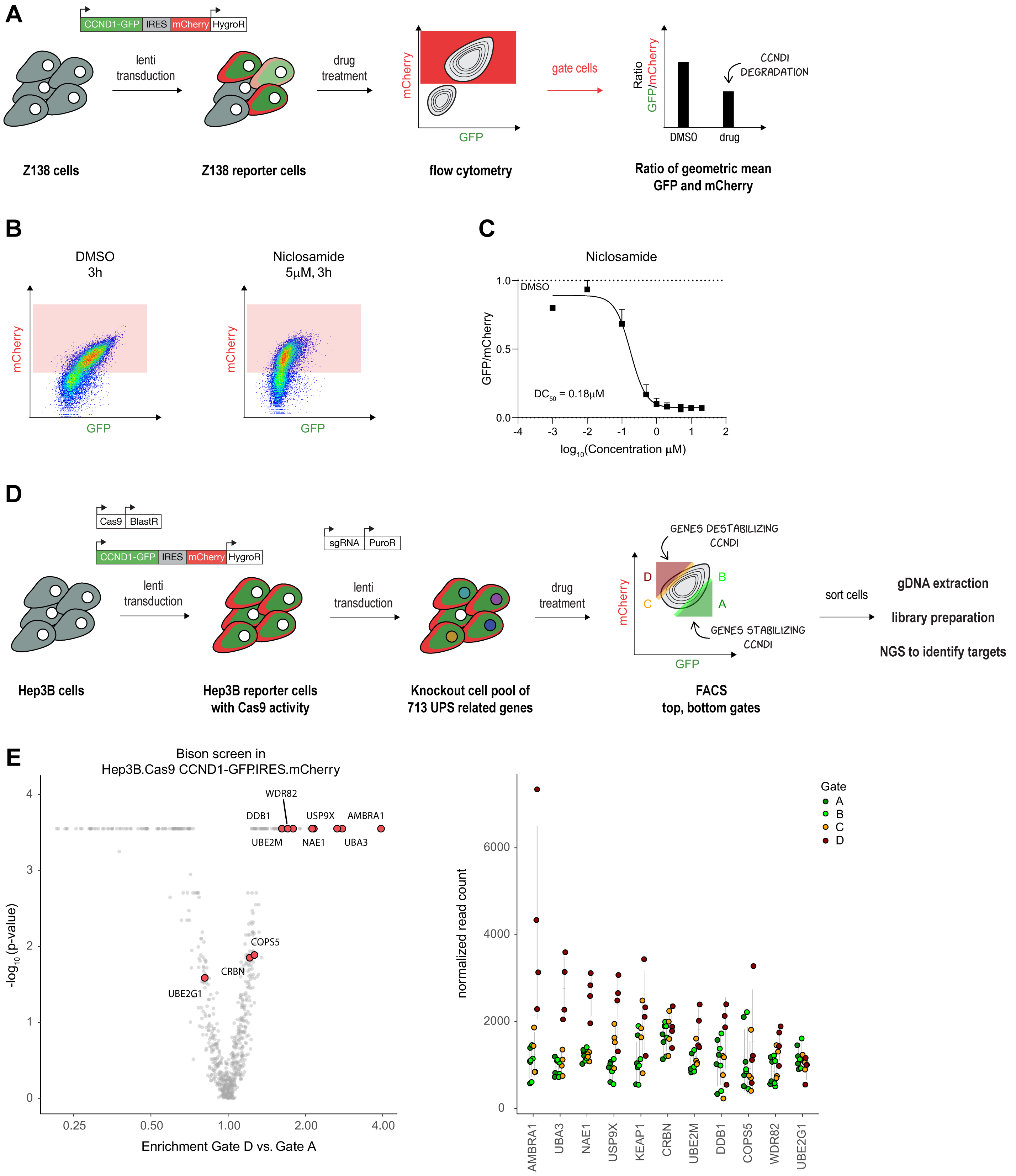
A
CCND1-GFP IRES mCherry HygroR
lenti transduction
drug treatment
mCherry
GFP
gate cells
Ratio GFP/mCherry
CCND1 DEGRADATION
DMSO drug
Z138 cells
Z138 reporter cells
flow cytometry
Ratio of geometric mean GFP and mCherry
B
DMSO 3h
Niclosamide 5µM, 3h
mCherry
GFP
C
Niclosamide
DMSO
GFP/mCherry
DC50 = 0.18µM
log10(Concentration µM)
D
Cas9 BlastR
CCND1-GFP IRES mCherry HygroR
sgRNA PuroR
lenti transduction
lenti transduction
drug treatment
GENES DESTABILIZING CCND1
GENES STABILIZING CCND1
sort cells
gDNA extraction
library preparation
NGS to identify targets
Hep3B cells
Hep3B reporter cells with Cas9 activity
Knockout cell pool of 713 UPS related genes
FACS top, bottom gates
E
Bison screen in Hep3B.Cas9 CCND1-GFP.IRES.mCherry
WDR82
DDB1 USP9X AMBRA1
UBE2M NAE1 UBA3
COPS5
CRBN
UBE2G1
-log10 (p-value)
Enrichment Gate D vs. Gate A
normalized read count
Gate
A
B
C
D
AMBRA1 UBA3 NAE1 USP9X KEAP1 CRBN UBE2M DDB1 COPS5 WDR82 UBE2G1

A

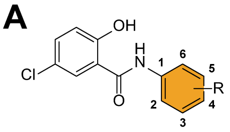

2-Cl, 4-NO_2	**Niclosamide**
2-Cl, 4-NH_2	**1a**
2-Cl, 4-H	**1b**
2-Cl, 4-CN	**1c**
2-Cl, 4-CO_2Me	**1d**
2-F, 4-NO_2	**1e**
3-CF_3, 5-CF_3	**1f**
2-Cl, 4-SO_2NH_2	**1g**
2-H, 4-NO_2	**1h**
2-Cl, 4-CF_3	**1i**
2-H, 5-*t*Bu	**1j**
2-Cl, 4-F	**1k**
2-Cl, 4-Cl	**1l**
2-NO_2, 4-Cl	**1m**
3-Cl, 5-NO_2	**1n**

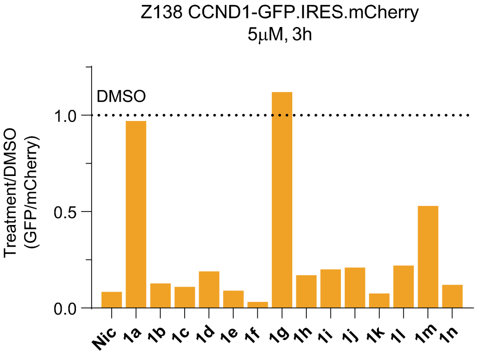

B

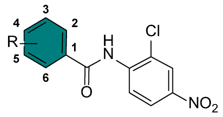

2-OH, 5-Cl	**Niclosamide**
2-OBz, 5-Cl	**2a**
2-Cl, 5-OH	**2b**
2-NH_2, 5-Cl	**2c**
2-OH, 4-Cl	**2d**
2-Cl, 4-OH	**2e**
2-OH, 6-Cl	**2f**
2-OH, 5-F	**2g**
2-Br, 5-Cl	**2h**
2-H, 5-Cl	**2i**
2-OH, 5-H	**2j**

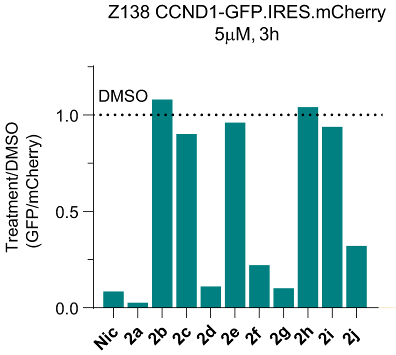

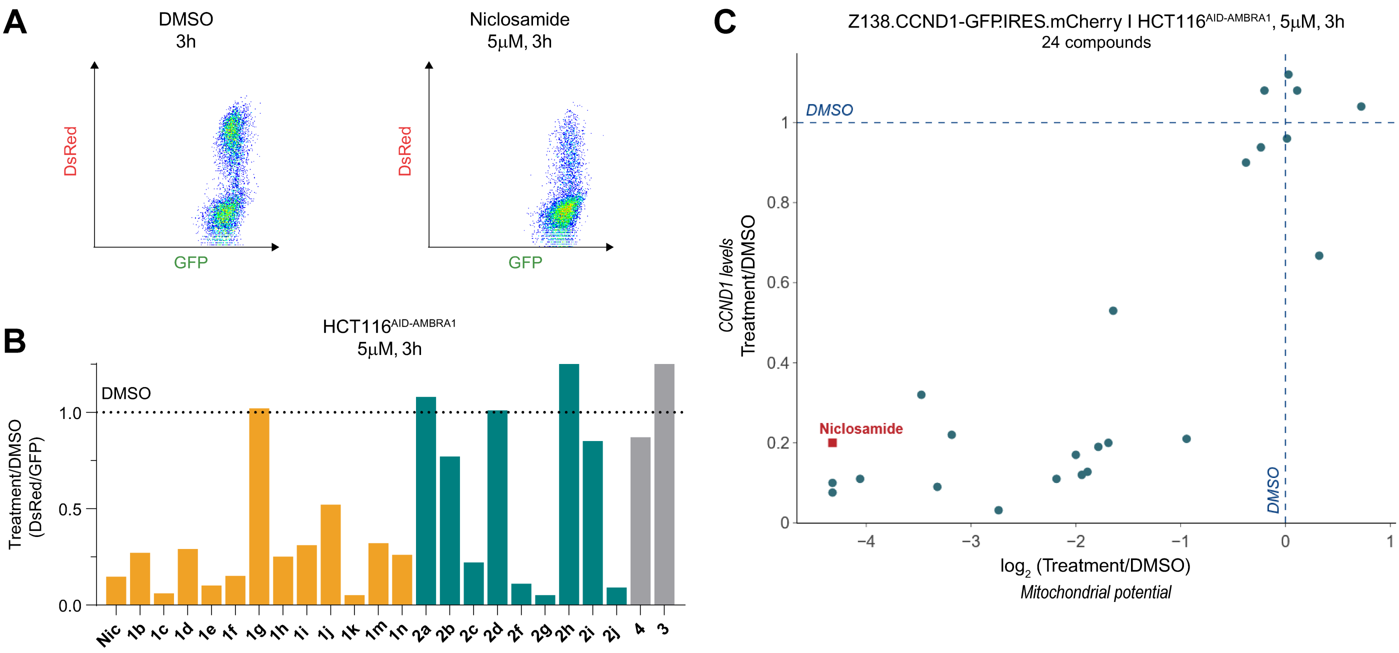
A
DMSO
3h
Niclosamide
5µM, 3h
DsRed
GFP
B
HCT116AID-AMBRA1
5µM, 3h
Treatment/DMSO
(DsRed/GFP)
DMSO
1.0
0.5
0.0
Nic 1b 1c 1d 1e 1f 1g 1h 1i 1j 1k 1m 1n 2a 2b 2c 2d 2f 2g 2h 2i 2j 4 3
C
Z138.CCND1-GFP.IRES.mCherry I HCT116AID-AMBRA1, 5µM, 3h
24 compounds
CCND1 levels
Treatment/DMSO
DMSO
Niclosamide
log2 (Treatment/DMSO)
Mitochondrial potential
1
0.8
0.6
0.4
0.2
0
−4
−3
−2
−1
0
1

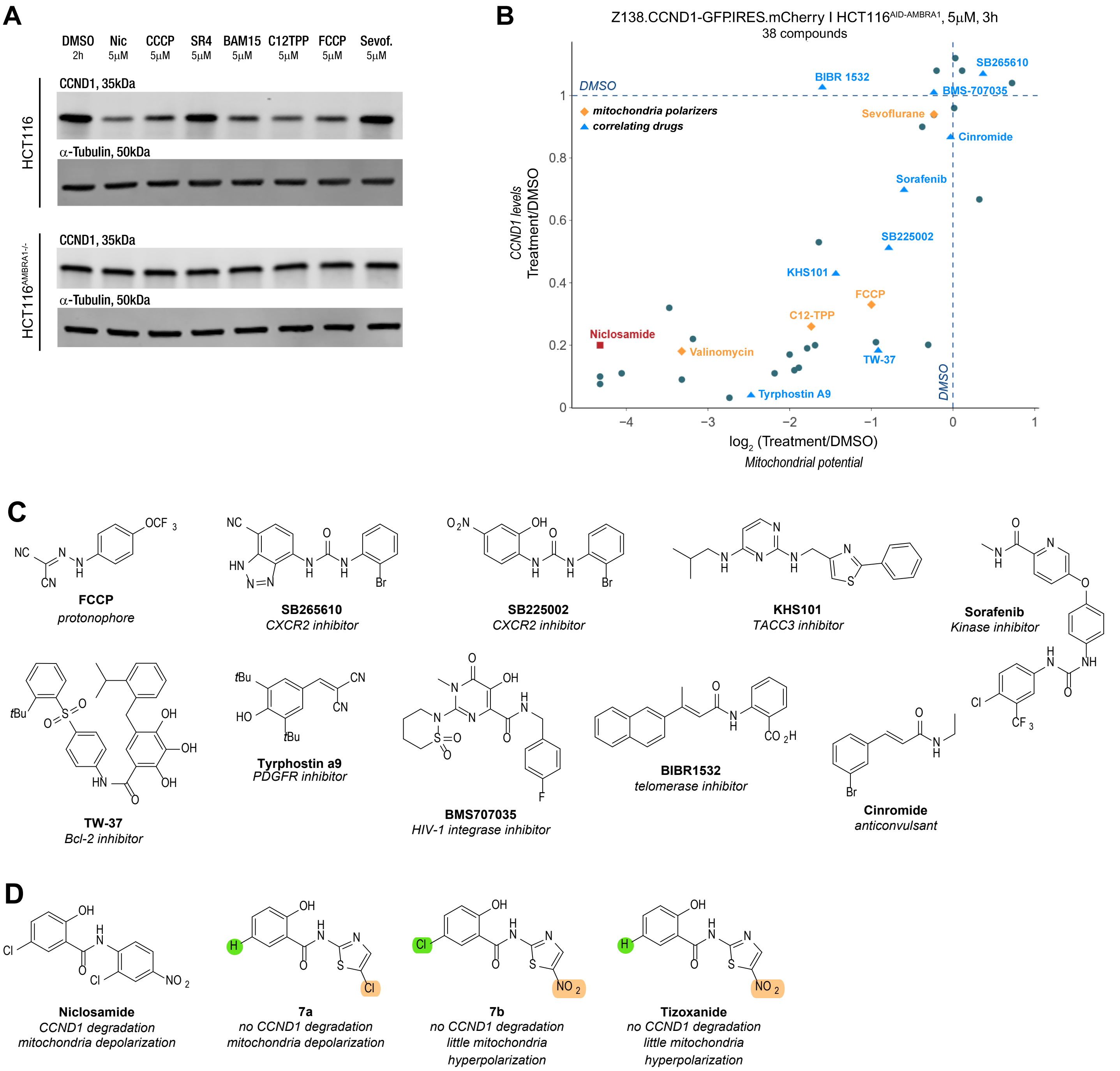
A
DMSO 2h
Nic 5µM
CCCP 5µM
SR4 5µM
BAM15 5µM
C12TPP 5µM
FCCP 5µM
Sevof. 5µM
HCT116
CCND1, 35kDa
α-Tubulin, 50kDa
HCT116AMBRA1-/-
CCND1, 35kDa
α-Tubulin, 50kDa
B
Z138.CCND1-GFPIRES.mCherry I HCT116AID-AMBRA1, 5µM, 3h
38 compounds
DMSO
mitochondria polarizers
correlating drugs
CCND1 levels
Treatment/DMSO
log2 (Treatment/DMSO)
Mitochondrial potential
SB265610
BIBR 1532
BMS-707035
Sevoflurane
Cinromide
Sorafenib
SB225002
KHS101
FCCP
C12-TPP
Niclosamide
Valinomycin
TW-37
Tyrphostin A9
C
FCCP
protonophore
SB265610
CXCR2 inhibitor
SB225002
CXCR2 inhibitor
KHS101
TACC3 inhibitor
Sorafenib
Kinase inhibitor
TW-37
Bcl-2 inhibitor
Tyrphostin a9
PDGFR inhibitor
BMS707035
HIV-1 integrase inhibitor
BIBR1532
telomerase inhibitor
Cinromide
anticonvulsant
D
Niclosamide
CCND1 degradation
mitochondria depolarization
7a
no CCND1 degradation
mitochondria depolarization
7b
no CCND1 degradation
little mitochondria
hyperpolarization
Tizoxanide
no CCND1 degradation
little mitochondria
hyperpolarization

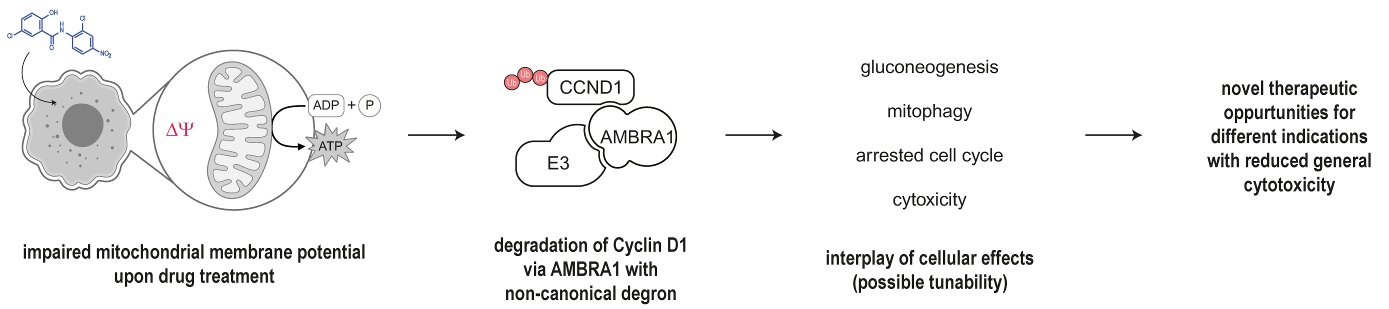
ADP
P
ATP
ΔΨ
impaired mitochondrial membrane potential upon drug treatment
CCND1
AMBRA1
E3
degradation of Cyclin D1 via AMBRA1 with non-canonical degron
gluconeogenesis
mitophagy
arrested cell cycle
cytoxicity
interplay of cellular effects (possible tunability)
novel therapeutic oppurtunities for different indications with reduced general cytotoxicity

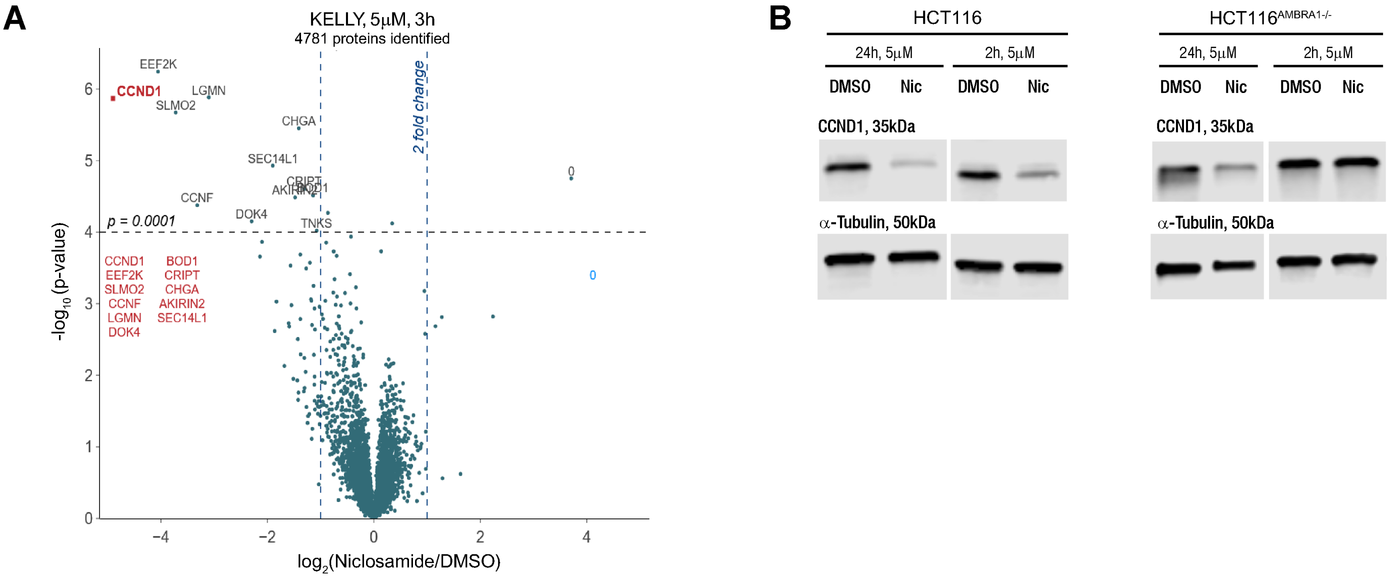
A
KELLY, 5µM, 3h
4781 proteins identified
EEF2K
CCND1
LGMN
SLMO2
CHGA
SEC14L1
CRIPT
AKIRIN2
BOD1
CCNF
DOK4
TNKS
2 fold change
p = 0.0001
CCND1
EEF2K
SLMO2
CCNF
LGMN
DOK4
BOD1
CRIPT
CHGA
AKIRIN2
SEC14L1
0
0
-log10 (p-value)
log2(Niclosamide/DMSO)
B
HCT116
24h, 5µM
2h, 5µM
DMSO
Nic
DMSO
Nic
CCND1, 35kDa
α-Tubulin, 50kDa
HCT116AMBRA1-/-
24h, 5µM
2h, 5µM
DMSO
Nic
DMSO
Nic
CCND1, 35kDa
α-Tubulin, 50kDa

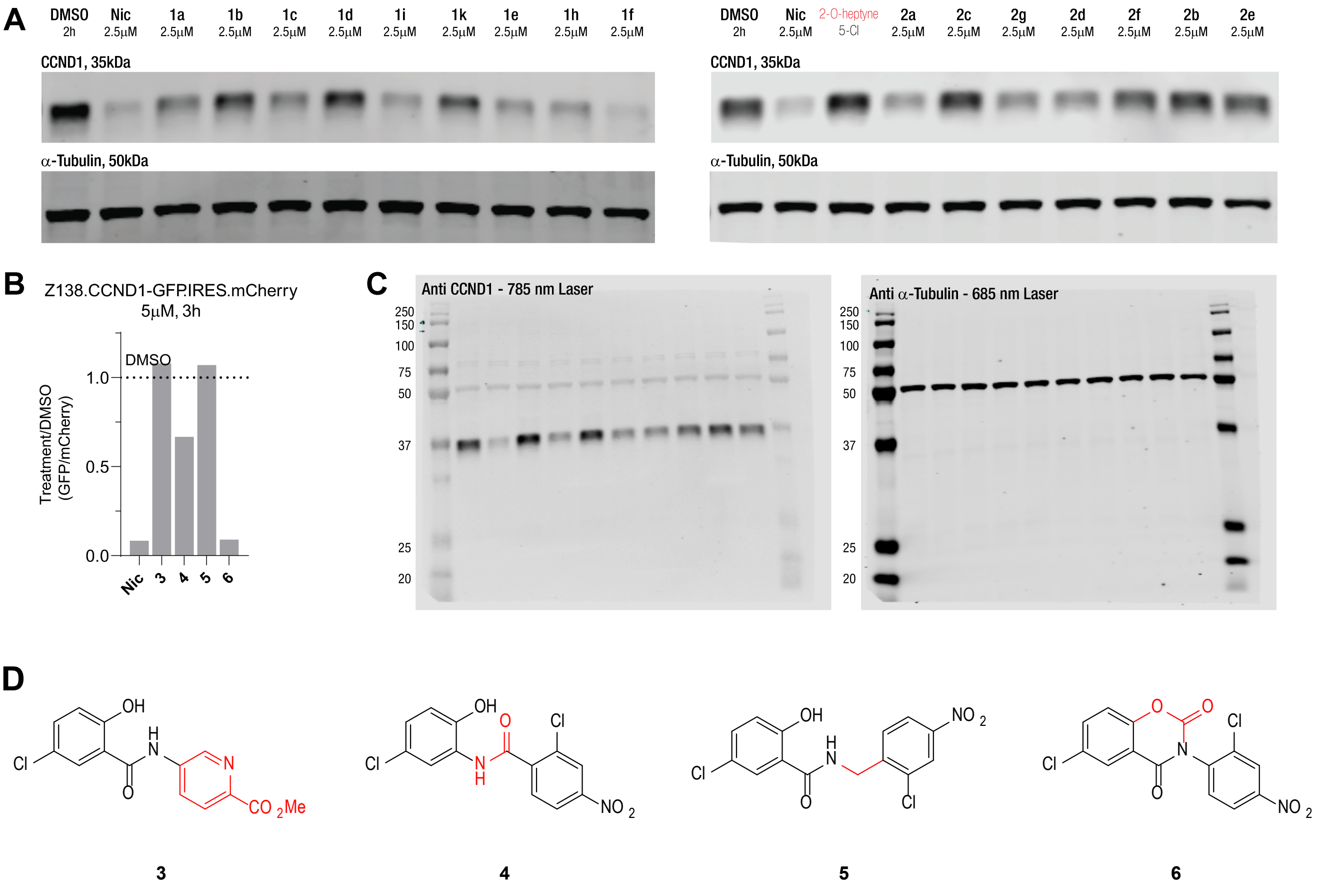

A
DMSO 2h
Nic 2.5μM
1a 2.5μM
1b 2.5μM
1c 2.5μM
1d 2.5μM
1i 2.5μM
1k 2.5μM
1e 2.5μM
1h 2.5μM
1f 2.5μM
CCND1, 35kDa
α-Tubulin, 50kDa
DMSO 2h
Nic 2.5μM
2-O-heptyne 5-Cl
2a 2.5μM
2c 2.5μM
2g 2.5μM
2d 2.5μM
2f 2.5μM
2b 2.5μM
2e 2.5μM
CCND1, 35kDa
α-Tubulin, 50kDa
B
Z138.CCND1-GFP.IRES.mCherry
5μM, 3h
Treatment/DMSO (GFP/mCherry)
DMSO
1.0
0.5
0.0
Nic
3
4
5
6
C
Anti CCND1 - 785 nm Laser
250
150
100
75
50
37
25
20
Anti α-Tubulin - 685 nm Laser
250
150
100
75
50
37
25
20
D
3
4
5
6

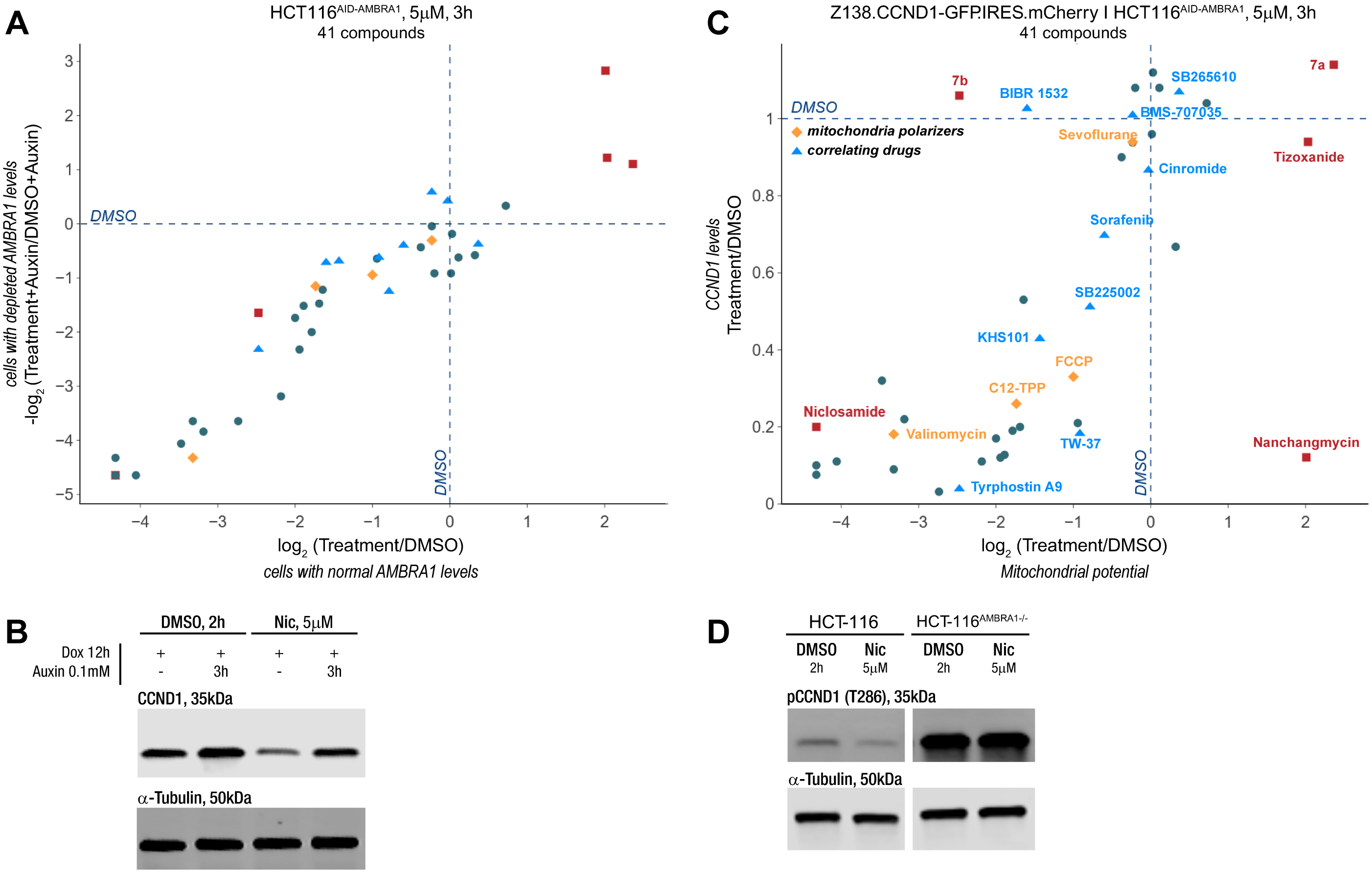

E

MACCS fingerprint

	Niclosamide	FCCP	Valinomycin	Tyrphostin A9	TW-37	KHS101	SB225002	Sorafenib
Niclosamide	1	0.35	0.344262295	0.229508197	0.493150685	0.179487179	0.807017544	0.492307692
FCCP	0.35	1	0.211538462	0.333333333	0.232876712	0.216666667	0.35483871	0.418181818
Valinomycin	0.344262295	0.211538462	1	0.295454545	0.378787879	0.213114754	0.328125	0.436363636
Tyrphostin A9	0.229508197	0.333333333	0.295454545	1	0.292307692	0.155172414	0.21875	0.263157895
TW-37	0.493150685	0.232876712	0.378787879	0.292307692	1	0.231707317	0.435897436	0.376623377
KHS101	0.179487179	0.216666667	0.213114754	0.155172414	0.231707317	1	0.1875	0.308823529
SB225002	0.807017544	0.35483871	0.328125	0.21875	0.435897436	0.1875	1	0.538461538
Sorafenib	0.492307692	0.418181818	0.436363636	0.263157895	0.376623377	0.308823529	0.538461538	1

Morgan fingerprint

	Niclosamide	FCCP	Valinomycin	Tyrphostin A9	TW-37	KHS101	SB225002	Sorafenib
Niclosamide	1	0.139784946	0.036144578	0.11	0.207142857	0.133928571	0.455696203	0.247863248
FCCP	0.139784946	1	0.008064516	0.157303371	0.110344828	0.091743119	0.126315789	0.232142857
Valinomycin	0.036144578	0.008064516	1	0.040816327	0.079310345	0.038314176	0.027777778	0.046931408
Tyrphostin A9	0.11	0.157303371	0.040816327	1	0.169014085	0.068965517	0.098039216	0.091603053
TW-37	0.207142857	0.110344828	0.079310345	0.169014085	1	0.181818182	0.214285714	0.264150943
KHS101	0.133928571	0.091743119	0.038314176	0.068965517	0.181818182	1	0.196261682	0.152173913
SB225002	0.455696203	0.126315789	0.027777778	0.098039216	0.214285714	0.196261682	1	0.235294118
Sorafenib	0.247863248	0.232142857	0.046931408	0.091603053	0.264150943	0.152173913	0.235294118	1
