# Supplementary material for: Niclosamide: CRL4AMBRA1 mediated degradation of cyclin D1 following mitochondrial membrane depolarization
Source: RSC Med Chem. 2025 May 6. Online ahead of print. doi: 10.1039/d5md00054h (PMC12054360; doi:10.1039/d5md00054h)

Supplementary Information (SI) for RSC Medicinal Chemistry.
This journal is © The Royal Society of Chemistry 2025

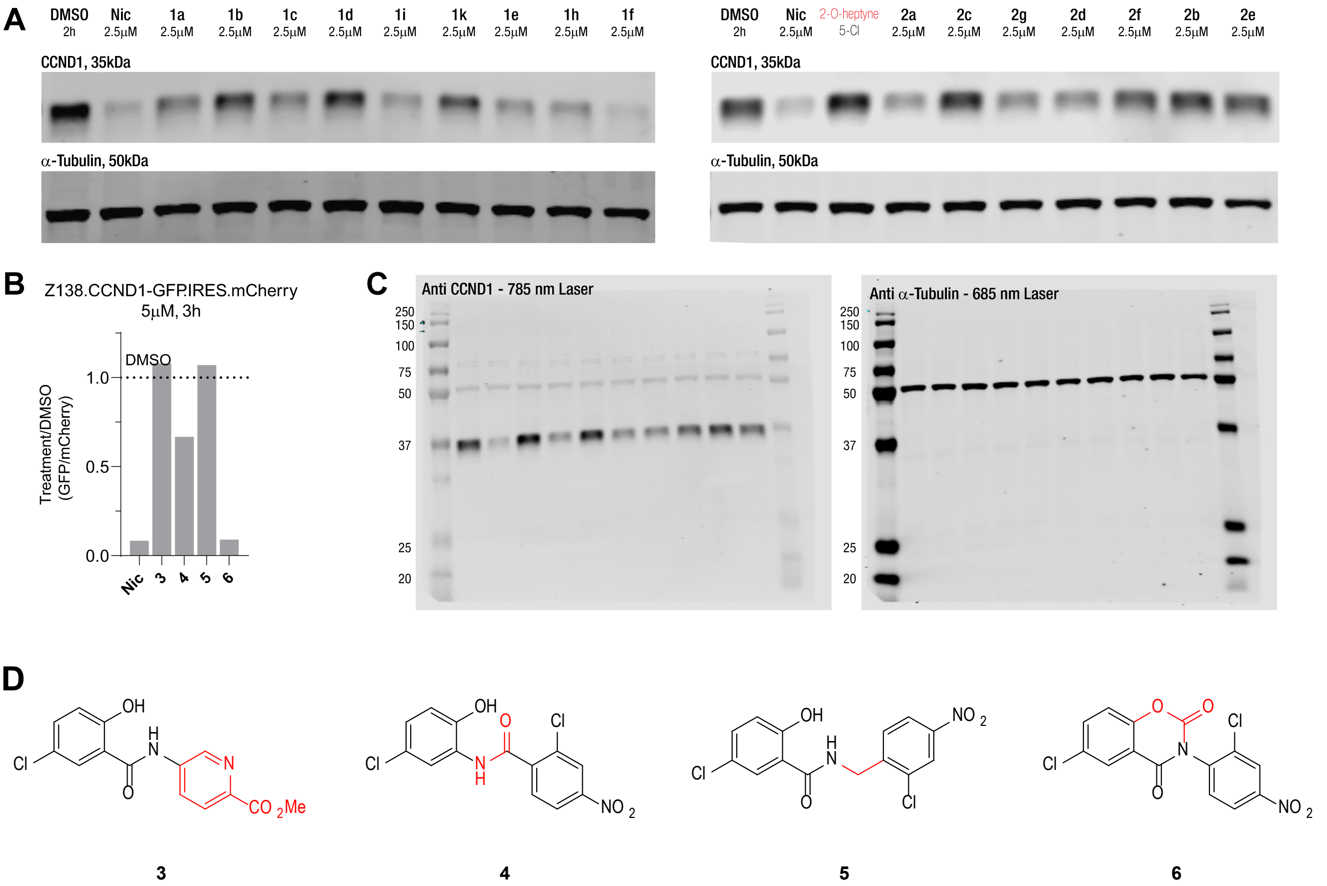

Supplement: Supplementary file 2 [file MD-OLF-D5MD00054H-s003.pdf]
