# Supplementary material for: Niclosamide: CRL4AMBRA1 mediated degradation of cyclin D1 following mitochondrial membrane depolarization
Source: RSC Med Chem. 2025 May 6. Online ahead of print. doi: 10.1039/d5md00054h (PMC12054360; doi:10.1039/d5md00054h)
Supplement: Supplementary file 3 [file MD-OLF-D5MD00054H-s004.pdf]

Supplementary Information (SI) for RSC Medicinal Chemistry.
This journal is © The Royal Society of Chemistry 2025

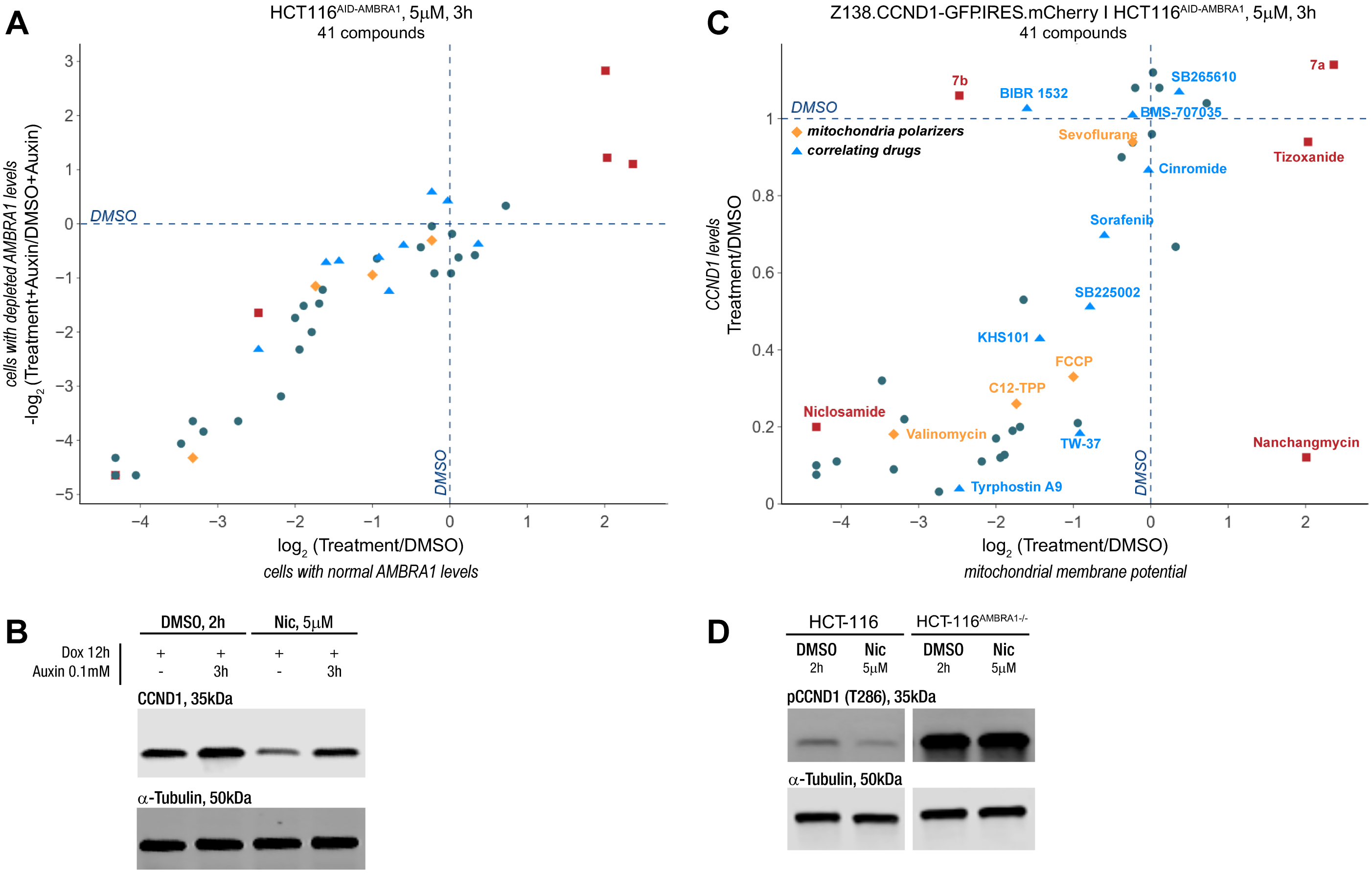

E

MACCS fingerprint

	Niclosamide	FCCP	Valinomycin	Tyrphostin A9	TW-37	KHS101	SB225002	Sorafenib
Niclosamide	1	0.35	0.344262295	0.229508197	0.493150685	0.179487179	0.807017544	0.492307692
FCCP	0.35	1	0.211538462	0.333333333	0.232876712	0.216666667	0.35483871	0.418181818
Valinomycin	0.344262295	0.211538462	1	0.295454545	0.378787879	0.213114754	0.328125	0.436363636
Tyrphostin A9	0.229508197	0.333333333	0.295454545	1	0.292307692	0.155172414	0.21875	0.263157895
TW-37	0.493150685	0.232876712	0.378787879	0.292307692	1	0.231707317	0.435897436	0.376623377
KHS101	0.179487179	0.216666667	0.213114754	0.155172414	0.231707317	1	0.1875	0.308823529
SB225002	0.807017544	0.35483871	0.328125	0.21875	0.435897436	0.1875	1	0.538461538
Sorafenib	0.492307692	0.418181818	0.436363636	0.263157895	0.376623377	0.308823529	0.538461538	1

Morgan fingerprint

	Niclosamide	FCCP	Valinomycin	Tyrphostin A9	TW-37	KHS101	SB225002	Sorafenib
Niclosamide	1	0.139784946	0.036144578	0.11	0.207142857	0.133928571	0.455696203	0.247863248
FCCP	0.139784946	1	0.008064516	0.157303371	0.110344828	0.091743119	0.126315789	0.232142857
Valinomycin	0.036144578	0.008064516	1	0.040816327	0.079310345	0.038314176	0.027777778	0.046931408
Tyrphostin A9	0.11	0.157303371	0.040816327	1	0.169014085	0.068965517	0.098039216	0.091603053
TW-37	0.207142857	0.110344828	0.079310345	0.169014085	1	0.181818182	0.214285714	0.264150943
KHS101	0.133928571	0.091743119	0.038314176	0.068965517	0.181818182	1	0.196261682	0.152173913
SB225002	0.455696203	0.126315789	0.027777778	0.098039216	0.214285714	0.196261682	1	0.235294118
Sorafenib	0.247863248	0.232142857	0.046931408	0.091603053	0.264150943	0.152173913	0.235294118	1
